# Combination of Antidepressants and Chemotherapeutic Agents to Overcome P-Glycoprotein-Mediated Resistance in Cancer Patients: A Systematic Review

**DOI:** 10.3390/medsci14010126

**Published:** 2026-03-07

**Authors:** Antonio Restaino, Mario Pinto, Giulio Carriero, Antonio Maria D’Onofrio, Silvia Montanari, Delfina Janiri, Giovanni Camardese, Lorenzo Moccia, Gabriele Sani, Alessio Simonetti

**Affiliations:** 1 Section of Psychiatry, Department of Neuroscience, Università Cattolica del Sacro Cuore, 00168 Rome, Italy; mario.pinto@guest.policlinicogemelli.it (M.P.);; 2 Section of Psychiatry, Department of Neuroscience, Head-Neck and Chest, Fondazione Policlinico Universitario Agostino Gemelli IRCCS, 00168 Rome, Italy; g.camardese@unilink.it; 3 Department of Life Science, Health, and Health Professions, Link Campus University, 00165 Rome, Italy; 4 Department of Psychiatry and Behavioral Sciences, Baylor College of Medicine, Houston, TX 77030, USA

**Keywords:** P-glycoprotein, multidrug resistance, multidrug resistance reversal, antidepressants, chemosensitization, chemotherapy

## Abstract

**Background/Objectives:** P-glycoprotein (P-gp, ABCB1/MDR1) is a key ATP-binding cassette transporter involved in multidrug resistance in cancer, limiting intracellular accumulation of various chemotherapeutic (CT) agents. Several antidepressants (ADs) have been shown to modulate P-gp function. This dual pharmacological profile raises the possibility of repurposing ADs as chemosensitizers to enhance anticancer drug efficacy. The objective of this review was to summarize the available evidence on the combined use of ADs and chemotherapeutics to overcome P-gp-mediated resistance. **Methods:** A systematic search was performed in PubMed, Scopus, and PsycInfo/PsycArticles databases using a comprehensive search string combining terms for P-gp, ADs, chemotherapy, and drug resistance. Inclusion criteria were preclinical or clinical studies investigating the effect of ADs in combination with chemotherapeutics on P-gp-mediated resistance in cancer models. Eleven relevant studies were identified and qualitatively analyzed. **Results:** Across diverse cancer models, including colon, breast, and multidrug-resistant cell lines, several ADs significantly enhanced the cytotoxicity of many chemotherapeutic agents. The proposed mechanisms involved downregulation of P-gp expression, inhibition of efflux activity, and increased intracellular drug accumulation. **Conclusions:** The combination of ADs with CT agents shows promising potential in overcoming P-gp-mediated multidrug resistance, enhancing antitumor efficacy in preclinical models. Further translational and clinical research is needed to validate these findings, optimize dosing strategies, and assess the risk–benefit profile in cancer patients, particularly those with comorbid depressive disorders.

## 1. Introduction

Cancer is a massive burden on global health systems. It causes about 16% of all deaths across the world and contributes to roughly 25% of deaths from noncommunicable diseases. In adults between 30 and 69 years of age, it drives close to one-third of early mortality and consistently appears among the top three causes of death in the vast majority of countries [1].

Advances in cytotoxic chemotherapy, targeted therapies, immunotherapy, and supportive care have significantly improved survival rates across several malignancies [2,3,4]. Nevertheless, the long-term efficacy of anticancer treatments continues to be undermined by the emergence of multidrug resistance (MDR) [5].

MDR is a major biological obstacle to effective cancer therapy and a leading cause of treatment failure. It is defined as the ability of cancer cells to exhibit resistance to multiple structurally and mechanistically unrelated anticancer agents and may be present at baseline as intrinsic resistance or may develop during treatment as acquired resistance under therapeutic selective pressure [6]. Key contributors include P-glycoprotein (P-gp), Multidrug Resistance Protein 1 (MRP-1), and Breast Cancer Resistance Protein (BCRP), all parts of the ATP-Binding Cassette (ABC) transporter superfamily, which comprises 48 ATP-dependent efflux pumps [7,8]. These proteins are physiologically expressed in multiple tissues—including the adrenal cortex, placenta, gastrointestinal and hepatobiliary tracts, renal tubules, and the blood–brain barrier—where they normally expel lipids, peptides, and toxic substrates from the cytoplasm [9]. In cancer, however, these cells hijack this process to expel chemotherapeutic (CT) agents, thereby undermining intracellular drug accumulation and compromising therapeutic efficacy. The overexpression of P-gp represents one of the most extensively characterized and clinically relevant pathways [10] involved in MDR. P-gp is frequently overexpressed in cancer cells, where it recognizes CT agents as toxic substances and expels them, reducing their intracellular concentration and therapeutic effect [10]. This mechanism compromises the efficacy of many cytotoxic drugs and even affects some newer targeted agents. Consequently, inhibiting P-gp has emerged as a potential strategy to overcome MDR, as it is considered a key transporter driving chemotherapy resistance in several carcinomas [4,5,7,10,11].

Recent preclinical evidence has suggested that certain pharmacological agents not traditionally employed in oncology may modulate P-gp activity [12,13]. Among them, some ADs have been studied [14,15,16]. This pharmacodynamic interaction raises the possibility that combining ADs with standard CT regimens could enhance intracellular drug retention, restore chemosensitivity, and ultimately improve antitumor efficacy.

On this basis, the therapeutic repurposing of ADs as adjuvants to chemotherapy offers a compelling and biologically rational strategy to counteract P-gp–mediated resistance. The present systematic review examines the available evidence supporting this approach, exploring whether the concomitant administration of ADs and CT agents can effectively overcome P-gp–driven drug resistance and enhance treatment response in cancer models.

## 2. Materials and Methods

### Search Strategy

A search strategy on PubMed, Scopus, and PsycInfo/PsycArticles databases was performed to identify all literature published before 1 October 2025. The search was conducted by two researchers. The complete search string employed in this review is reported in the Supplementary Materials (Section S1).

Abstracts were screened according to inclusion and exclusion criteria. Papers included in this review met the following criteria: (i) published in any language, at any time; (ii) original research articles (reviews and meta-analyses were excluded, although their reference lists were screened to identify additional eligible studies); (iii) conducted on in vitro tumor cell lines, in vivo animal models, or adult cancer patients (≥18 years of age); (iv) investigated the combination of AD with CT agents; (v) explicitly assessed outcomes related to P-glycoprotein (P-gp)-mediated resistance, including P-gp expression or activity, modulation of drug efflux, chemosensitivity, cytotoxicity, tumor response, or survival outcomes. Exclusion criteria were as follows: (i) reviews, meta-analyses, consensus meetings, or guidelines (collectively labeled as “Review”); (ii) editorials, commentaries, letters to the editor, and opinion papers lacking original data (collectively labeled as “Editorial”); (iii) study protocols (“Protocol”); (iv) corrigenda, errata, or other correction notices not containing original data (“Corrigendum”); (v) retracted publications, regardless of study type (“Retracted”); (vi) articles not peer-reviewed or with an ongoing peer-review process (“Preprint”); (vii) studies not involving cancer-related models or patients and studies evaluating ADs or chemotherapeutics alone, without combination (“Unfocused”); (viii) case reports or case series lacking systematic methodology (“Case”); (ix) studies involving participants under 18 years of age (“Lumping”); (x) duplicate publications across databases (“Duplicate”). All studies that met the inclusion criteria were categorized as “Included.”

**Figure 1:**
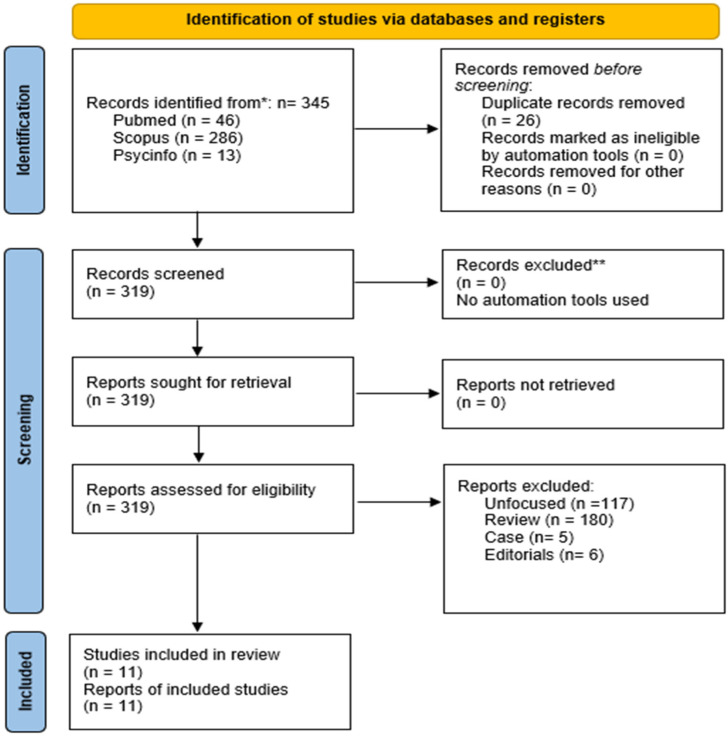
Flowchart of the systematic literature search according to PRISMA guidelines. * Consider, if feasible to do so, reporting the number of records identified from each database or register searched (rather than the total number across all databases/registers). ** If automation tools were used, indicate how many records were excluded by a human and how many were excluded by automation tools.

Inclusion and exclusion decisions were reached through unanimous author consensus, established via iterative Delphi rounds. Two rounds were sufficient to reach complete agreement for paper inclusion or exclusion (Supplementary Materials, Table S1–S3). This review was performed in accordance with the PRISMA (Preferred Reporting Items for Systematic Reviews and Meta-Analyses) guidelines (Figure 1 and Supplementary Materials, Table S4) [17]. The PRISMA checklist and flowchart, as well as detailed results of database searches, are provided in the Supplementary Materials. The methodological quality and Risk of Bias (RoB) of the included studies were assessed using tools specifically designed for preclinical in vitro and in vivo research. In vitro studies were evaluated using the Office of Health Assessment and Translation (OHAT) RoB Tool, which examines key domains such as the adequacy of exposure selection and characterization, blinding of outcome assessors, completeness of outcome data, and the presence of selective reporting [18]. For in vivo animal studies, the Systematic Review Centre for Laboratory Animal Experimentation (SYRCLE) RoB Tool was applied. This instrument assesses multiple methodological domains, including sequence generation, allocation concealment, blinding during experimental procedures and outcome assessment, management of incomplete outcome data, and selective outcome reporting [19]. Any disagreement arising during the quality assessment process was resolved through discussion and consensus among the reviewers.

We registered our review on Open Science Framework (OSF), URL https://osf.io/97pz3 (accessed on 23 December 2025). Overall judgments and reviewer comments for each rated study are provided in the Supplementary Materials (Tables S1–S3). The methodological quality and RoB of the included studies are summarized in the Supplementary Materials (Table S5, Figures S1 and S2). The full list of references for all included studies is available in the Supplementary Material (Table S6).

## 3. Results

The systematic search identified 345 records in total, with 46 retrieved from PubMed, 286 from CINAHL, and 13 from PsycInfo. Following the application of the exclusion criteria, 334 records were discarded. Eleven studies satisfied the inclusion criteria and were considered for qualitative synthesis (see Table 1 and Table 2). The full list of the included studies in chronological order is presented in the Supplementary Materials (Table S6). A qualitative overview of the included studies is provided in the Supplementary Materials (Section S2).

### 3.1. Quality Assessment

The OHAT RoB Rating Tool [18] was used to evaluate the RoB of the included studies (see Supplementary File, Table S5 and Figure S1). Questions 3 and 4 of the OHAT tool are not applicable to in vitro studies and were therefore excluded from the assessment, although the original numbering of the OHAT domains was retained for consistency.

The overall RoB was generally low. In the Selection domain, randomization of exposure (Question 1) was rated as probably high risk in eight studies; probably low risk in three. Allocation concealment (Question 2) was consistently judged as high risk across all studies, reflecting the absence of concealment procedures in in vitro designs.

For the Performance domain, uniformity of experimental conditions (Question 5) was predominantly rated as very low (*n* = 7) or low risk (*n* = 4), highlighting the controlled nature of laboratory protocols. Conversely, blinding of research personnel (Question 6) was uniformly rated as high risk across all studies, which is expected in in vitro experiments where blinding is rarely feasible.

The Attrition domain showed excellent methodological quality: all studies provided complete outcome data, with eight judged as very low risk and three as low risk (Question 7). The Detection domain exhibited similarly positive results. For exposure characterization (Question 8), most studies were probably low (*n* = 7) or definitely low risk (*n* = 4). For outcome assessment (Question 9), six studies were rated as very low risk, five as low risk, and none as high risk.

Regarding Selective Reporting (Question 10), the distribution reflected strong transparency, with six studies evaluated as very low risk and five as low risk. The Other Sources of Bias domain (Question 11) was likewise reassuring: six studies were rated as very low risk and four as low risk, while only one study received a probably high-risk judgment due to limited reporting of methodological or analytical procedures. Overall, the in vitro evidence base demonstrated a predominantly low to very low RoB across most OHAT domains. The only consistently high-risk finding concerned the lack of blinding of study personnel, which represents an inherent and expected limitation of in vitro laboratory research rather than a methodological flaw.

The RoB for the in vivo studies was assessed using the SYRCLE RoB tool [19] (see Supplementary File, Table S7 and Figure S2). Studies 1, 2, 5, 6, and 11 did not include an in vivo component and were, therefore, not evaluated with SYRCLE. Based on the remaining studies, the distribution of ratings indicates that most domains were predominantly assessed as low risk or unclear risk, while high-risk judgments were infrequent and limited to specific items. In the Selection domain, both sequence generation and baseline characteristics showed consistently strong performance, with five to six studies rated as low risk, and no study rated as high risk. Allocation concealment, however, remained poorly reported, yielding five unclear ratings and one study classified as high risk.

Within the Performance domain, random housing and performance blinding showed mixed reporting, with the majority of studies judged as unclear. Nevertheless, one study achieved low risk for each of these items, and only two studies were rated as high risk for blinding of personnel.

The Detection domain demonstrated a similar pattern: random outcome assessment and outcome assessment blinding were predominantly unclear (five studies each), but one study in each domain was assessed as low risk.

In the Attrition and Selective Reporting domains, all six studies were rated as low risk for incomplete outcome data and for selective outcome reporting, indicating complete datasets and transparent reporting across the in vivo evidence base.

In the Other Sources of Bias domain, five studies were rated as low risk, with only one study classified as unclear and none as high risk.

Overall, despite some common reporting gaps typical of preclinical animal studies, the RoB assessment indicated that the methodological quality of the in vivo evidence was generally robust.

### 3.2. Aims of the Included Studies

A subset of investigations explicitly targeted P-gp as a primary mechanistic endpoint, aiming to determine whether ADs modulate this efflux transporter and thereby reverse multidrug resistance. This group includes Szabó et al. (1999) [20], which benchmarked ADs against cyclosporine A as an ABC-transporter inhibitor; Palmeira et al. (2011) [21], which used pharmacophore-based screening to identify ADs as P-gp modulators; Zhang et al. (2013) [22] and Özkaya Gül et al. (2025) [23], which quantified P-gp expression changes associated with SSRI-induced chemosensitization; and Wang et al. (2025) [24], which incorporated P-gp suppression into a platinum-based hybrid design to overcome cisplatin resistance. In all these studies, P-gp modulation was a declared and central component of the research objectives.

A second cluster of studies evaluated P-gp as a secondary or supportive endpoint, measuring transporter expression or efflux activity to contextualize broader aims related to chemosensitization, intracellular drug accumulation, apoptotic signaling, or pharmacokinetic enhancement [21,24,25]. In these works, P-gp was not specified in the stated aims, but was nonetheless assessed to interpret the mechanisms underlying restored drug sensitivity or modulation of MDR phenotypes.

Fan et al. (1992) [26] did not identify P-gp as an a priori study objective. This study was designed to determine whether ADs could reverse multidrug resistance, without prespecifying drug-efflux transporters as mechanistic endpoints. Nonetheless, ancillary experiments assessing P-gp expression and recognition through photoaffinity binding were performed.

### 3.3. Main Findings

Across the twelve preclinical investigations, ADs consistently potentiated the activity of multiple CT agents against MDR tumor models by increasing intracellular drug accumulation, reversing transporter-mediated efflux, and reinforcing apoptosis, while exhibiting minimal intrinsic cytotoxicity at chemosensitizing concentrations.

Early experimental work provided proof-of-concept that non-cytotoxic doses of TCAs can dismantle MDR phenotypes. Fan et al. (1992) [26] showed that Amoxapine, Imipramine, Maprotiline, Mianserine, Nortriptyline, and Protriptyline enhanced adriamycin-mediated growth inhibition in murine fibrosarcoma cells; notably, Trazodone increased intracellular adriamycin and vinblastine levels in low-P-gp parental cells without affecting P-gp expression or binding, pointing to chemosensitization mechanisms not solely dependent on ABC transporter blockade.

Szabó et al. (1999) [20] showed that Amitriptyline, Maprotiline, Trimipramine, Desipramine, Imipramine, and Doxepin increased R-123 and daunorubicin accumulation in MDR carcinoma and leukemia cell lines, while sparing drug-sensitive counterparts. Importantly, these effects replicated ex vivo in peripheral blood lymphocytes from patients with AML, where ADs restored daunorubicin accumulation to levels similar to cyclosporine A, demonstrating clinically relevant ABC transporter inhibition.

Computational and mechanistic validation was later provided by Palmeira et al. (2011) [21], who employed pharmacophore-based virtual screening to identify ADs as candidate P-gp modulators. Amoxapine emerged as a potent non-competitive P-gp inhibitor, significantly increasing R-123 accumulation, inhibiting ATPase activity, and reducing the doxorubicin GI_50_ by ≈3.5-fold in K562Dox cells.

In vitro–in vivo translation with SSRIs was first demonstrated by Peer et al. (2004) [27]. In MDR cell lines, fluoxetine increased the cytotoxic activity of multiple chemotherapeutics by 10- to 100-fold, driven by enhanced intracellular drug accumulation and reduced efflux. In vivo, fluoxetine elevated doxorubicin intratumoral concentrations by ~12-fold without altering its systemic pharmacokinetics. The combination of fluoxetine and doxorubicin yielded 2- to 3-fold improvements in tumor responses and survival, reversing MDR at doses far below clinically approved exposure levels and without added systemic toxicity. Argov et al. (2008) [28] corroborated these findings, showing that fluoxetine reduced doxorubicin IC_50_ by approximately 10-fold, increased intracellular drug accumulation (+32%), and inhibited efflux (−70%) in moderately resistant HCT-15 colorectal cancer cells. In vivo, the fluoxetine–doxorubicin combination significantly slowed tumor growth to a degree comparable to bevacizumab, despite requiring far fewer administrations. These findings confirm that SSRI-mediated chemosensitization extends to colorectal cancer models expressing moderate P-gp–dependent resistance.

Subsequent mechanistic studies refined the biological scope of SSRI-induced chemosensitization. Zhang et al. (2013) [22] showed that fluoxetine increased adriamycin and paclitaxel cytotoxicity in MCF-7/ADR breast cancer cells by downregulating P-gp, GST-π, and MDR1 mRNA expression. Liu et al. (2017) [29] demonstrated that fluoxetine enhanced cisplatin efficacy in cervical cancer cells and xenografts by suppressing anti-apoptotic proteins (Bcl-2) and upregulating caspase-9, consistent with apoptosis induction.

Other SSRIs and psychoactive agents showed comparable effects. Drinberg et al. (2014) [30] reported that sertraline reduced doxorubicin efflux by ≈3-fold, increased intracellular drug accumulation, and improved survival in xenografted mice treated with pegylated liposomal doxorubicin. In Duarte et al. (2022) [25], P-gp was assessed immunohistochemically. In MCF-7 breast cancer cells, the combination of paclitaxel and fluoxetine reduced the proportion of P-gp–positive cells compared with paclitaxel alone, whereas no P-gp expression was detected in HT-29 colorectal cancer cells. The study did not include functional efflux assays; P-gp modulation was observed at the expression level only, without mechanistic evidence of transporter inhibition.

Recent work extended these findings to lung cancer. Özkaya Gül et al. (2025) [23] demonstrated that escitalopram synergizes with etoposide at sub-IC_50_ levels, reducing P-gp expression, increasing PTEN and caspase-3, and selectively killing resistant A549 cells while sparing bronchial epithelial controls.

Wang et al. (2025) [24] synthesized fluoxetine-conjugated Pt(IV) prodrugs that markedly increased intracellular platinum accumulation, DNA platination, and both apoptosis and autophagy. In cisplatin-resistant A549 cells, these hybrids downregulated multiple resistance determinants, including P-gp, GST-π, ATM, and RAD51, overcoming chemoresistance. Moreover, in murine triple-negative breast cancer models, the lead compounds outperformed cisplatin in tumor control while reducing systemic toxicity and enhancing antitumor immune features, such as increased CD4^+^/CD8^+^ infiltration and immunogenic cell death markers.

**Table 1:** Summary of clinical characteristics of selected studies.

Study	Design	Population	Antidepressant(s)	Dosage	Chemoterapeutic Agent(s)	Dosage	Method/Technique Used
**Fan et al., 1992 [26]**	In vitro	UV-2237M murine fibrosarcoma parental line (ADR-sensitive) and MDR variant UV-2237M (ADR-resistant)	AMO, IMI, MAP, MIA, NOR, PRO, TRZ	0.1 µg/mL in combination assays; cytotoxicity profiled at 0.1, 1, and 10 µg/mL; TRZ pretreatment at 0.1 µg/mL for 16–24 h in accumulation assays	ADR (DOX), VBL, VCR, ACD, MMC, 5-FU	(IC_50_, µg/mL) ADR Parent: 0.88 ± 0.22 Parent + TRZ: 0.08 ± 0.02 MDR: 10.7 ± 0.4 MDR + TRZ: 7.4 ± 0.5 VBL Parent: 0.0025 ± 0.0009 Parent + TRZ: 0.0011 ± 0.0004 MDR: 0.052 ± 0.010 MDR + TRZ: 0.036 ± 0.008 VCR Parent: 0.015 ± 0.003 Parent + TRZ: 0.009 ± 0.0001 MDR: 0.23 ± 0.06 MDR + TRZ: 0.15 ± 0.05 5-FU Parent: 0.058 ± 0.001 Parent + TRZ: 0.059 ± 0.003 MDR: 0.068 ± 0.007 MDR + TRZ: 0.072 ± 0.008	Cytostasis (MTT assay), cytolysis ([^125^I]IdUrd), drug accumulation ([^14^C]ADR, [^3^H]VBL), P-gp expression (FITC-C219 flow cytometry), photoaffinity labeling ([^3^H]azidopine), PKC activity assay, calmodulin assay
**Szabó et al., 1999 [20]**	In vitro + ex vivo	Human PBL from cancer pts; human leukemia cell lines (L1210, L5178, KB-3-1) and corresponding MDR1-transfected resistant variants (L5178 MDR, KB-V-1). Three pts with AML.	AMI, MAP, TRI, DES, IMI, and DXP	Each AD was tested at concentrations of 0.5, 1, and 5 µg/mL.	DR, CYT, DOX, TAD/COAP	First pt: 45 mg/m^2^ of DR for 3 days; 100 mg/m^2^ of CYT for 7 days. Second pt: 45 mg/m^2^ of DOX for 3 days; 10 mg/m^2^ of CYT for 7 days. Third pt: 45 mg/m^2^ of DR for 3 days; 100 mg/m^2^ of CYT for 7 days.	[^125^I]IdUrd DNA synthesis assay; trypan blue viability; R-123 and [^3^H]-DR accumulation/efflux by flow cytometry; P-gp detection with FITC-C219 monoclonal antibody; [^3^H]-azidopine photoaffinity labeling to characterize P-gp binding sites; PKC and calmodulin pathway exclusion assays.
**Peer et al., 2004 [27]**	In vitro + in vivo	HT29 (human colon carcinoma), P388/WT (murine leukemia), MCF-7 (human breast carcinoma), C-26 (Colon carcinoma), B16F10.9 (highly aggressive mouse melanoma), D122 (highly aggressive mouse Lewis lung carcinoma), P388/ADR (derived from P388/WT, murine leukemia cells) and MCF-7/ADR (derived from MCF-7, human breast carcinoma)	FLX	5–15 µmol/L in vitro. 0.04 mg/kg body/day in vivo	VBL, MMC, DOX, PTX, CDDP.	(IC_50_, µM) MMC/MMC + FLX MCF-7: 30/28 P388/WT: 45/42 HT29: 30/33 B16F10.9: 209/3.6 D122: 209/3.3 C-26: 105/2.5 MCF-7/ADR: 200/4.5 P388/ADR: 224/5.4 DOX/DOX + FLXMCF-7: 0.35/0.33 P388/WT: 4.7/4.8 HT29: 3.6/3.3 B16F10.9: 12/0.78 D122: 19/0.9 C-26: 25/0.84 MCF-7/ADR: 19/0.29 P388/ADR: 31/0.55 VBL/VBL + FLX MCF-7: 0.28/0.30 P388/WT: 4.2/4.4 HT29: 3.5/3.3 B16F10.9: 13/0.45 D122: 21/1 C-26: 9.1/0.25 MCF-7/ADR: 22/0.32 P388/ADR: 38/1.4	Cell viability assays: MTT (metabolic activity), Trypan blue (membrane integrity), Bradford protein assay (total protein). Flow cytometry: R-123 accumulation to assess P-gp activity. Drug efflux/accumulation studies: DOX, PTX, VBL (including radiolabeled tracers). Plasma curves and tissue distribution of DOX. In vivo tumor models: measurement of tumor volume, metastatic burden, and survival in syngeneic and xenograft mice.
**Argov et al., 2008 [28]**	In vitro + in vivo	HCT-15 (in vitro); nude mice bearing HCT-15 xenografts (in vivo).	FLX	10–25 µM in vitro; 1 mg/kg orally in vivo	DOX, BEV	In vitro (IC_50_ values, µM): DXR 0.1–30; 10 for accumulation and efflux assays; FLX 10 for sensitization; 25 µM during accumulation/efflux assays; BEV 0.1–10. In vivo: DXR: 2 mg/kg i.v (3 doses); FLX: 1 mg/kg/day (6 times per week for 3 weeks). BEV: 5 mg/kg (5 times per week for 3 weeks).	XTT cytotoxicity assay; flow cytometry for P-gp, MRP, and BCRP expression; DOX accumulation and efflux assays; confocal microscopy for intracellular DOX distribution; in vivo tumor volume measurement and survival monitoring.
**Palmeira et al., 2011 [21]**	In silico + in vitro	Human cell lines: K562 (chronic myelogenous leukemia) and K562Dox (DOX-resistant, P-gp overexpressing	AMO	Tested at 10 µM and 20 µM (R-123 accumulation); 10 µM in combination with DOX (SRB assay); 200 µM in the ATPase assay	DOX	Variable, with determination of GI50 (≈11.6 µM alone; reduced to ~3.3 µM when combined with AMO).	In silico pharmacophore modeling and DrugBank virtual screening to identify AD-like structures as putative P-gp inhibitors; Western blotting to confirm P-gp overexpression and exclude MRP-1 involvement; R-123 retention by flow cytometry to quantify P-gp–mediated efflux; P-gp ATPase activity assays to define the inhibition mechanism; SRB cytotoxicity assay in K562Dox cells to determine DOX chemosensitization; PCA to cluster candidate compounds based on shared pharmacophoric features.
**Zhang et al., 2013 [22]**	In vitro	Human breast cancer cell line resistance MCF-7/ADM; MCF-7	FLX	5 µg/mL	ADR (DOX) PTX	(IC50, mg/mL) ADR/ADR + FLX: 13.62/2.71 in MCF-7/ADM; 0.53/0.45 in MCF-7. PTX/PTX + FLX: 3.3/2.59 in MCF-7/ADM; 2.11/1.22 in MCF-7.	Cell viability assay (MTT); Western blotting (P-gp, GST-π); RT-PCR and SYBR Green–based qRT-PCR (MDR1 mRNA)
**Drinberg et al., 2014 [30]**	Longitudinal, in vitro + in vivo study	OVCAR-8 and NCI/ADR-RES human ovarian cancer cell lines (in vitro); nude mice with NCI/ADR-RES xenografts (in vivo).	SER	10–15 µM in vitro; 2 mg/kg p.o. in vivo	DOX, DOXIL	DOX in vitro: 10 µM; in vivo: 2 mg/kg i.v., administered every other day for a total of 12 doses DOXIL in vivo: 2 mg/kg i.v., administered every three days for a total of 12 doses.	XTT cytotoxicity assay; flow cytometry for P-gp, MRP1, and BCRP expression; drug efflux assays using fluorescent substrates (R-123 and DOX) to assess intracellular accumulation and extrusion; quantitative analysis of intracellular DOX fluorescence; in vivo xenograft tumor implantation; tumor volume measurement and monitoring.
**Liu et al., 2017 [29]**	In vitro + in vivo study	Human cervical cancer cells HeLa	FLX	In vitro: 1–256 μM; In vivo: 1 mg/kg per day	CDDP	In vitro: 1–256 μM; In vivo: 3 mg/kg per day	FACS analysis; Apoptosis assay: detection by Annexin V-FITC/PI; samples analyzed by flow cytometry. Western blot analysis: proteins extracted with RIPA lysis buffer; quantified by BCA assay; separated by SDS-PAGE and transferred to PVDF membranes. Visualization by ECL
**Duarte et al., 2022 [25]**	In vitro	MRC-5 human normal lung fibroblast cell line, MCF-7 human breast adenocarcinoma cells, and HT-29 (in vitro).	FLX, SER	IC50 (µM) FLX 6.12 in HT-29; 7.7 in MCF-7; SER 2.45 in HT-29; 2.22 in MCF-7	PTX, 5-FU	DOX 0.17 µM in MCF-7; PTX 2.78 nM in MCF-7; 5-FU 3 µM in HT-29.	MTT cytotoxicity assay; CMA construction followed by immunocytochemistry to evaluate protein expression (PPT1, P-gp/MRP1, MRP2, Ki67, cleaved-PARP, NF-kB p65) in MCF-7 and HT-29 cells after drug treatments; evaluation of staining pattern (nuclear, cytoplasmic, or membrane) and percentage of positive cells.
**Wang et al., 2025 [24]**	In vitro + in vivo	Human: HepG2, HeLa, HCT-116, A549, MCF-7 breast adenocarcinoma cells, MDA-MB-231, A549cisR and LO2 (in vitro). BALB/c mice with 4T1-Luc syngeneic breast cancer model and BALB/c nude mice with MDA-MB-231 tumor xenografts (in vivo).	FLX–PT(IV) prodrug	FLX not administered as a free drug; delivered exclusively as a Pt(IV) prodrug.	CDDP, OXP	FLX–Pt(IV) prodrug: 2 mg-Pt/kg i.p. every 2 days, follow-up 3 mg-Pt/kg i.p. every 2 days CDDP: 2 mg/kg i.p. every 2 days OXP: 5 mg/kg i.p. every 2 days	MTT antiproliferative assay; cellular uptake analysis of platinum compounds using ICP-MS; apoptosis evaluation using Annexin V-FITC/PI staining and flow cytometry; wound healing assay to assess cell migration; transwell invasion assay with Matrigel-coated plates; immunofluorescence assay for protein localization; western blot for protein expression quantification; assessment of ROS content with DCFH-DA fluorescent probe; mitochondrial membrane potential analysis using JC-1; R-123 accumulation assay in A549cisR cells; autophagosome formation assay using MDC; autophagy flux analysis with BafA1 and LC3 immunofluorescence/Western blot; Syngeneic and xenograft mouse models for in vivo antitumor therapy, safety, and efficacy evaluation; small animal bioluminescence imaging for tumor growth and metastasis monitoring; tumor volume measurement (V = L × W^2^ × 0.5); H&E staining and immunohistochemistry for post-mortem tissue analysis.
**Özkaya Gül et al., 2025 [23]**	In vitro	A549 (NSCLC), A549/90E (etoposide-resistant NSCLC), BEAS-2B (normal bronchial epithelial)	ES	ES used at 0.5×, 1×, and 2× IC_50_ for each cell line; IC_50_ values (mg/mL): A549 = 1.84, A549/90E = 5.49, BEAS-2B = 2.92.	ET	ET used at 0.5×, 1×, 2× IC_50_ for each cell line; IC_50_ values (µg/mL): A549 = 48.67, A549/90E = 499.82, BEAS-2B = 79.38.	CK-8, trypan blue, neutral red assays; Annexin V-FITC/PI; JC-1 (ΔΨm); ELISA (caspase-3, PTEN, P-gP); CompuSyn synergy analysis; PPI network analysis

Abbreviations: ΔΨm, Mitochondrial Membrane Potential (Delta Psi m); [^125^I]IdUrd, [^125^I]-iododeoxyuridine; 4T1-Luc, Luciferase-labeled mouse breast cancer cells; 5-FU, 5-Fluorouracil; A549, non-small-cell lung carcinoma cells; A549cisR, cisplatin-resistant non-small-cell lung carcinoma cells; ACD, Actinomycin D; AD, Antidepressant; ADR, Adriamycin; AMI, Amitriptyline; AML, acute myeloid leukemia; AMO, Amoxapine: BafA1, Bafilomycin A1; BCA, Bicinchoninic Acid assay; BCRP, breast cancer resistance protein; BEV, Bevacizumab; CCK-8, Cell Counting Kit-8; CDDP, cisplatin; CMA, cell microarray; CYT, Citarabine; DCFH-DA, 2′,7′-dichlorodihydrofluorescein diacetate; DES, Desipramine; DOX, doxorubicin; DOXIL, pegylated liposomal doxorubicin; DR, daunorubicin; DXP, Doxepin; ELISA, Enzyme-Linked Immunosorbent Assay; ECL, Enhanced ChemiLuminescence; ES, Escitalopram oxalate; ET, Etoposide; FACS, Fluorescence-Activated Cell Sorting; FITC, fluorescein isothiocyanate; FLX, Fluoxetine; GI50, Growth Inhibition 50; HCT-, human colorectal cancer cell line-; H&E, hematoxylin and eosin; HeLa, epithelial cervical carcinoma cells; HepG2, hepatocellular carcinoma cells; HT-29; human colorectal adenocarcinoma cell line; IC50, Inhibitory concentration 50; ICP-MS, inductively coupled plasma mass spectrometry; IMI, Imipramine; i.v., intravenous; JC-1, 5,5′,6,6′-Tetrachloro-1,1′,3,3′-tetraethylimidacarbocyanine iodide; KB-3-1, heLa-derived cervical cancer subclone, sensitive; LC3, microtubule-association protein 1 light chain-3 protein; LO2, normal hepatic cells; MAP, Maprotiline; MCF, Michigan Cancer Foundation; MDA-MB-231; triple-negative breast carcinoma cells-231; MDC, monodansylcadaverine; MDR, Multidrug Resistance; MIA, Mianserine; MMC, Mitomycin C; MRC, Medical Research Council; MRP, multidrug resistance-associated proteins; MTT, thiazolyl blue tetrazolium bromide; mRNA, messenger RNA; NCI/ADR-RES, National Cancer Institute/Adriamycin-Resistant; NF-kB, nuclear factor-kappa-B; NOR, Nortriptyline; NSCLC, Non-Small Cell Lung Cancer; OVCAR-8, Ovarian Carcinoma-line 8; OXP, oxaliplatin; p.o., per os; P-gp; P-glycoprotein; PARP, poly(ADP-ribose) polymerase; PBL, peripheral blood lymphocytes; PCA, Principal Component Analysis; PI, propidium iodide; p.o., per os; PPI, Protein–Protein Interaction; PPT1, palmitoyl-protein thioesterase 1; PRO, Protriptyline: pt(s), patients(s); PT, Platinum, PTEN, Phosphatase and Tensin Homolog; PTX, Paclitaxel; PVDF, PolyVinylidene DiFluoride; qRT-PCR, quantitative Reverse Transcription Polymerase Chain Reaction; R-123, Rhodamine-123; RIPA, RadioImmunoPrecipitation Assay buffer; ROS, reactive oxygen species; RT-PCR, Reverse Transcription Polymerase Chain Reaction; SDS-PAGE, Sodium Dodecyl Sulfate—PolyAcrylamide Gel Electrophoresis; SER, Sertraline; SRB, Sulforhodamine B assay; TAD/COAP, Tioguanin-Alexan-Daunorubicin/Cyclophosphamide-Vincristine-Alexan-Prednisolone; TRI, Trimipramine; TRZ, Trazodone; VBL, Vinblastine; VCR, Vincristine; WT, wild-type: XTT, methoxynitrosulfophenyl-tetrazolium carboxanilide.

**Table 2:** Summary of aims and findings of selected studies.

Study	Aims	Main Findings
**Fan et al., 1992** [26]	To evaluate whether ADs modulate MDR-related resistance and clarify mechanisms	All ADs enhanced ADR-mediated cytostasis in parental cells; TRZ was the most effective, also sensitizing to VBL and VCR but not to ACD, MMC, or 5-FU. TRZ increased intracellular ADR and VBL in parental cells without altering P-gp expression or binding, nor PKC or calmodulin activity. Potential to reverse intrinsic resistance in low P-gp–expressing cells.
**Szabó et al., 1999** [20]	To investigate whether clinically used AD drugs can modulate P-gp mediated efflux of CT agents (DR) and fluorescent substrates (R-123) in MDR leukemia and carcinoma cell lines (L1210 MDR, L5178 MDR, KB-V-1) and in PBL from drug-resistant AML pts. The study aimed to determine the potential of AD drugs to enhance intracellular uptake of chemotherapeutics.	In MDR cell lines (L1210 MDR, L5178 MDR, KB-V-1), all tested ADs increased intracellular accumulation of R-123 or DR. No significant effect was observed in parental, drug-sensitive cell lines (L1210, L5178, KB-3-1). In ex vivo PBL from AML pts, two out of three pts showed enhanced uptake of DR comparable to the effect of the known P-gp blocker CsA. The other pt did not respond to either CsA or AD drugs. Clinically relevant blood levels (10–500 ng/mL) of AD drugs produced modest but significant P-gp inhibition in cell lines; PBL could not be tested at the highest dose due to limited sample volume. Overall, the results suggest that AD drugs may sensitize MDR cells and patient-derived leukemic cells to CT agents, supporting the combination therapy.
**Peer et al., 2004** [27]	To determine whether FLX is an effective chemosensitizer in MDR cell cultures and in resistant mouse tumor models of both syngeneic and human xenografts, and to compare its efficacy, toxicity, and dosage with known chemosensitizers (verapamil, cyclosporine A).	In vitro, FLX increased the cytotoxic effects of multiple anticancer drugs in MDR cells by 10–100-fold, associated with increased intracellular drug accumulation and inhibition of efflux. In vivo, FLX augmented intratumoral DOX levels ~12-fold without altering its systemic pharmacokinetics. The FLX–DOX combination produced 2–3-fold improvements in tumor response and survival. MDR reversal occurred at doses well below established human safety limits and without the dose-dependent toxicity, adverse effects, or solubility issues typical of other chemosensitizers.
**Argov et al., 2008** [28]	To examine whether clinically used FLX can modulate P-gp–mediated efflux of the CT agent DOX in multidrug-resistant HCT-15 colorectal cancer cells and in vivo xenograft mouse models, while determining its potential to enhance intracellular uptake of DOX and sensitize resistant cancer cells to chemotherapy.	HCT-15 cells expressed P-gp, but not MRP or BCRP, showing moderate resistance to DOX (IC_50_ = 4.7 ± 0.3 µM). Combination with 10 µM FLX reduced IC_50_ about 10-fold (to 0.46 ± 0.04 µM), while FLX alone had no effect on cell viability. BEV had a negligible effect in vitro. 25 µM of FLX increased intracellular DOX accumulation by 32% and decreased efflux by 70%, indicating significant P-gp pump inhibition. In addition, FLX promoted nuclear accumulation of DOX, enhancing its cytotoxic effect compared to DOX alone. In vivo, three injections of DOX (cumulative dose 6 mg/kg) have minimally affected tumor growth. The combination of FLX (1 mg/kg p.o.) + DOX significantly slowed tumor progression (4-fold increase vs. 10–12 fold in other groups), achieving an effect comparable to BEV (15 injections, cumulative dose 75 mg/kg). Overall, FLX modulates MDR in vitro and in vivo at safe doses, supporting its potential use as a chemosensitizer in combination with DOX or another chemotherapeutic.
**Palmeira et al., 2011** [21]	To find new P-gp inhibitors among already known drugs using pharmacophore-based screening and lab validation.	Identified 21 candidate drugs, of which 12 inhibited P-gp (increased R-123 accumulation). Among them, AMO was identified as a potent non-competitive inhibitor of P-gp, significantly increasing R-123 accumulation and reducing the GI_50_ of DOX by ~3.5-fold in resistant K562Dox cells.
**Zhang et al., 2013** [22]	To research the effect of FLX on multidrug resistance mediated by P-gp and GST-π.	Pre-treatment with FLX enhances cytotoxicity significantly both on resistant and sensitive cells, downregulates the expression of P-gp and GST-*π* proteins in resistance cells, decreased the MDR1 mRNA by the FLX-PTX combination only. FLX-PTX combination reduced the P-gp expression in both MCF-7/ADR and MCF-7 cells. The FLX-ADR combination had no statistical significance but had the tendency to downregulate P-gp expression.
**Drinberg et al., 2014** [30]	To examine whether SER can act as a chemosensitizer by inhibiting P-gp–mediated efflux and reversing MDR in highly resistant OVCAR-8 and NCI/ADR-RES human ovarian cancer cell lines. To assess the effect of SER in combination with DOX and DOXI both in vitro and in vivo, and to determine whether combined therapy enhances intracellular DOX accumulation, cytotoxicity, and tumor regression compared to chemotherapy alone.	NCI/ADR-RES cells exhibited strong P-gp expression and moderate MRP1 expression, confirming their multidrug-resistant phenotype. SER (10–15 µM) significantly enhanced DOX cytotoxicity in resistant cells, reducing efflux about 3-fold and increasing intracellular drug accumulation, while showing no effect in sensitive OVCAR-8 cells. In vivo, combination therapy with DOX (2 mg/kg i.v.) and SER (2 mg/kg p.o.) slowed tumor growth and increased survival about 1.5-fold compared to DOX alone. The combination of DOXIL and SER further enhanced therapeutic efficacy, achieving greater tumor regression and 20% longer median survival versus DOXIL alone. Overall, results demonstrated that SER can effectively reverse MDR at low, safe doses by inhibiting efflux pumps and enhancing drug influx, suggesting a clinically translatable strategy for resistant ovarian cancers.
**Liu et al., 2017** [29]	To evaluate whether FLX can act as a chemosensitizer in CDDP-treated cervical cancer cells of the HeLa line. To analyze the effects of FLX alone and in combination with DDP on cell proliferation, cell cycle progression, and apoptosis, expression of genes associated with chemoresistance (P-gp, GST-π) and apoptosis (Bcl-2, caspase-9, p17). To validate in vivo the findings obtained in vitro, using HeLa xenograft tumor models in nude mice.	FLX in combination with DDP could significantly inhibit cellular proliferation. In vivo, FLX and DDP in combination effectively reduced the tumor weights. FLX and DDP combination significantly downregulated GST-π, P-gp, and Bcl-2 levels, and enhanced the expression of caspase-9 and p17.
**Duarte et al., 2022** [25]	To investigate the biosafety and mechanisms of action of combinations of antineoplastic drugs (DOX, PTX, 5-FU) with AD drugs (FLX, SER) in MCF-7 breast adenocarcinoma and HT-29 colon cancer cells, focusing on how these drug interactions affect cancer cell proliferation (Ki67), apoptosis (cleaved-PARP), autophagy and lysosomal function (PPT1), drug efflux (P-gp, MRP2), and NF-kB signaling, and whether the repurposed drugs enhance the anticancer efficacy of the chemotherapeutics.	In MCF-7 and HT-29 cancer cells, all tested AD drugs enhanced the effect of CT agents in combination treatments compared to single-agent exposure. PTX + FLX: combination reduced Ki67 proliferation marker and P-gp expression more effectively than PTX alone, with minimal cytotoxicity in non-tumoral MRC-5 fibroblasts at ≤2× IC_50_. PTX + SER: showed partial inhibition of PPT1 and P-gp, enhancing anticancer activity while sparing MRC-5 fibroblasts at sub-IC_50_ concentrations. 5-FU + Thioridazine: selectively reduced Ki67 proliferation marker in HT-29 cells and inhibited PPT1 more effectively than 5-FU alone, while maintaining normal cell viability in MRC-5 fibroblasts at ≤IC_50_; higher concentrations induced minor morphological changes. 5-FU + SER: combination decreased Ki67 and inhibited PPT1 in HT-29 cells, with minimal cytotoxicity in MRC-5 fibroblasts at ≤IC_50_; single-agent SER had little effect, and higher concentrations caused slight morphological alterations. Overall, AD drugs synergize with chemotherapeutics by reducing proliferation (Ki67) and modulating PPT1 and P-gp, improving anticancer efficacy while reducing toxicity in normal cells compared to single treatments.
**Wang et al., 2025** [24]	To design and synthesize a series of novel multifunctional Pt(IV) prodrug molecules by incorporating FLX into the Pt(IV) scaffold. To evaluate whether FLX-Pt(IV) complexes can enhance DNA damage, downregulate MDR-related proteins, and induce autophagic cell death in cancer cells. To investigate the anticancer efficacy and safety profile of the FLX-Pt(IV) prodrugs, leveraging the well-documented in vivo safety of FLX. To explore the potential of FLX-Pt(IV) complexes to exert multitarget, multipathway anticancer effects compared with traditional Pt(II)-based chemotherapy regimens.	FLX–Pt(IV) 8 and FLX–Pt(IV) 12 showed the strongest cytotoxicity, inducing greater DNA damage and apoptosis while maintaining selectivity toward tumor cells. Both compounds promoted S-phase arrest, increased intracellular platinum accumulation, and generated higher levels of DNA-bound platinum compared to CDDP. Treatment with FLX–Pt(IV) complexes led to increased ROS generation, mitochondrial dysfunction, and activation of autophagy, confirmed by LC3 and Beclin-1 upregulation with P62 downregulation. Autophagy induction occurred via eEF2K inhibition and AMPK–ULK1 pathway activation, suggesting a synergistic mechanism between the platinum center and the FLX moiety. In drug-resistant A549cisR cells, both compounds effectively overcame CDDP resistance, reducing the expression of MDR-related proteins (P-gp, GST-π, ATM, RAD51) and restoring sensitivity to platinum-based treatment. In vivo, FLX–Pt(IV) 8 and 12 significantly suppressed tumor growth in TNBC xenograft models, with higher tumor inhibition rates and markedly reduced systemic toxicity compared to CDDP. Both compounds preserved body weight and organ integrity, with no significant nephrotoxicity or hematologic toxicity observed. Moreover, FLX–Pt(IV) complexes enhanced antitumor immune responses, increasing CD4^+^ and CD8^+^ T-cell activation, promoting immunogenic cell death (CRT exposure, HMGB1 release), and downregulating PD-L1 expression in tumor tissue. Overall, FLX–Pt(IV) prodrugs demonstrated potent, multitarget anticancer activity through enhanced DNA damage, inhibition of eEF2K-mediated survival pathways, induction of autophagy and apoptosis, and immunomodulatory effects, achieving superior efficacy and safety compared to conventional Pt(II) chemotherapy.
**Özkaya Gül et al., 2025** [23]	To evaluate cytotoxic and apoptotic effects of ES alone and combined with ET, and assess the ability to overcome drug resistance	ES alone: cytotoxic in A549 and A549/90E, less toxic in BEAS-2B. ES + ET (½ IC50 each): synergistic in A549 and A549/90E, antagonistic in BEAS-2 B. Combination: increased caspase-3 and PTEN, decreased P-gP in resistant cells. Overall effect: selective apoptosis induction in cancer cells, sparing normal cells.

Abbreviations: 5-FU, 5-Fluorouracil; A549cisR, cisplatin-resistant non-small-cell lung carcinoma cells; ACD, Actinomycin D; AD(s), antidepressant(s); ADR, Adriamycin; AML, acute myeloid leukemia; AMO, Amoxapine; AMPK, AMP-activated protein kinase; ATM, Ataxia Telangiectasia Mutated; BCRP, breast cancer resistance protein; BEV, Bevacizumab; CD4^+^, Cluster of Differentiation 4 positive; CD8^+^, Cluster of Differentiation 8 positive; CDDP, cisplatin; CRT, calreticulin; CsA, cyclosporin A; CT, Chemoterapeutic; DOX, doxorubicin; DOXIL, pegylated liposomal doxorubicin; DR, daunorubicin; eEF2K, eukaryotic elongation factor-2 kinase; ES, Escitalopram; ET, Etoposide; FLX, Fluoxetine; GST-π, glutathione S-transferase π; HCT-, human colorectal cancer cell line-; HMGB1, high mobility group box 1 HT-29; human colorectal adenocarcinoma cell line; IC_50_, Half maximal inhibitory concentration; i.v., intravenous; LC3, microtubule-association protein 1 light chain-3 protein; MCF, Michigan Cancer Foundation; MDR, Multidrug Resistance; MMC, Mitomycin C; MRC, Medical Research Council; MRP, multidrug resistance-associated proteins; NCI/ADR-RES, National Cancer Institute/Adriamycin-Resistant; NF-kB, nuclear factor-kappa-B; OVCAR-8, Ovarian Carcinoma-line 8; P62, sequestosome 1; PARP, poly(ADP-ribose) polymerase; PBL, peripheral blood lymphocytes; PD-L1, programmed cell death-ligand 1; P-gp; P-glycoprotein; p.o., per os; PPT1, palmitoyl-protein thioesterase 1; pt(s), patient(s); Pt(II),platinum(II); Pt(IV), platinum(IV); PTX, Paclitaxel; R-123, Rhodamine-123; RAD51, DNA repair recombinase; ROS, reactive oxygen species; SER, Sertraline; SSRI, Serotonin Selective Reuptake Inhibitor; TNBC, triple-negative breast cancer; ULK1, TRZ, Trazodone; Unc-51 like autophagy activating kinase 1; VCR, Vincristine.

## 4. Discussion

**Figure 2:**
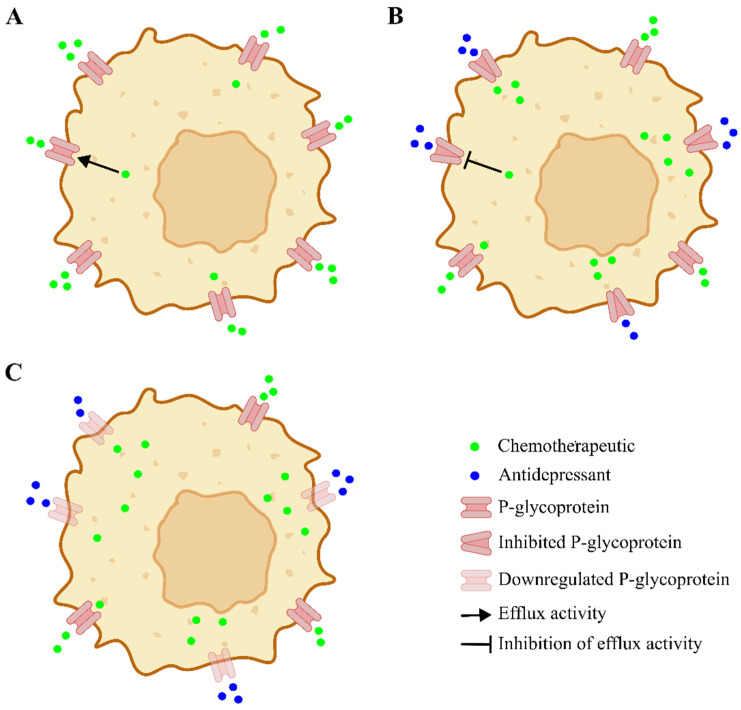
Schematic representation of AD-mediated modulation of P-gp activity. (**A**) Under baseline conditions, P-gp actively extrudes CT agents from the intracellular compartment, reducing intracellular drug accumulation and contributing to multidrug resistance. (**B**) In the presence of ADs, competitive inhibition of P-gp occurs through interaction with substrate-binding sites or functional interference with transporter activity, resulting in reduced efflux and increased intracellular retention of the chemotherapeutic agent. (**C**) ADs may additionally downregulate P-gp expression, leading to a decreased number of functional transporters at the cell membrane and a consequent reduction in overall efflux capacity, further enhancing intracellular chemotherapeutic accumulation.

Collectively, findings from the present systematic research indicate that ADs do not appear to exert a direct cytotoxic effect alone, but rather act as chemosensitizers across several tumors, enhancing the activity of anticancer agents in multidrug-resistant models while sparing drug-sensitive cells [20,21,22,23,26,27,28]. The predominant involved pathway is the modulation of drug efflux transporter, mainly P-gp. Chemosensitization is not a uniform class effect among ADs, but rather reflects agent-specific pharmacological properties and experimental contexts. Mechanisms underlying resistance reversal seem to involve both the functional inhibition of P-gp and the modulation of its expression [16,31] (see Figure 2). Some investigations [20,23,25,27,28,30] consistently reported increased intracellular accumulation of P-gp substrates such as R-123, DOX, and VBL, accompanied by reduced drug efflux. Notably, these effects frequently occurred in the absence of detectable changes in P-gp expression levels, suggesting that ADs primarily interfere with transporter activity rather than inducing rapid transcriptional or translational downregulation of MDR1. ADs might interfere with transporter activity, potentially through competitive interaction with substrate-binding sites, interference with ATPase activity, or alterations in membrane dynamics affecting transporter efficiency. In contrast, other studies have reported a direct modulation of P-gp expression. These effects may involve transcriptional or translational downregulation of MDR1, possibly secondary to enhanced cellular stress, activation of apoptotic pathways, or broader remodeling of survival and resistance-related signaling networks [32,33]. Specifically, AD–CT combinations have been shown to enhance apoptotic signaling, as evidenced by increased caspase activation and downregulation of anti-apoptotic proteins such as Bcl-225. In parallel, modulation of proliferation and survival markers, including Ki67, PARP cleavage, PPT1, and NF-κB signaling, has been described, suggesting a broader impact on tumor cell homeostasis [25,34,35]. Other investigations indicate that certain AD-based strategies induce mitochondrial dysfunction, oxidative stress, and autophagy, ultimately promoting immunogenic cell death [24,34,35,36]. Collectively, these findings suggest that ADs do not merely facilitate intracellular drug accumulation but may actively amplify downstream cell death pathways, with some formulations, such as fluoxetine-based Pt(IV) prodrugs, exhibiting true multitarget anticancer activity.

As regards the ADs investigated, fluoxetine emerges as the most extensively studied and consistently supported agent for chemosensitization [22,24,25,27,28,29]. Multiple independent studies have demonstrated its ability to enhance the cytotoxic efficacy of different CT agents across a wide range of tumor models, both in vitro and in vivo, often at clinically relevant concentrations, through inhibition of drug efflux and, in some settings, downregulation of P-gp expression.

Sertraline has also shown reproducible chemosensitizing activity, although it has been investigated in a more limited number of studies [25,30]. Available evidence supports its ability to reverse multidrug resistance primarily through functional inhibition of P-gp and increased intracellular drug accumulation, with consistent effects observed in both in vitro and in vivo models, particularly in ovarian and breast cancer settings.

Other ADs, such as amoxapine and amitriptyline, demonstrated potent P-gp–modulating effects mainly in comparative in vitro analyses, suggesting strong pharmacological activity but more limited translational validation to date [20,21,26].

A limited number of the included studies performed direct comparative analyses between ADs and non-AD compounds in the context of resistance reversal [20,21,25]. In these investigations, ADs were evaluated alongside a diverse range of pharmacological modulators, including calcium channel blockers (verapamil, diltiazem), antipsychotics (fluphenazine, haloperidol, loxapine, thioridazine), immunosuppressants (cyclosporin A), antifungal agents (econazole), and other repositioned drugs, providing a robust comparative framework for assessing resistance reversal. Notably, specific ADs emerged as particularly robust candidates across different experimental models: amoxapine has been ranked among the most potent P-gp modulators in comparative in vitro analyses [30], while amitriptyline [20], fluoxetine, and sertraline demonstrated consistent chemosensitizing effects in the other studies [20,25]. Unlike first- and second-generation P-gp inhibitors, whose development was limited by unacceptable toxicity and clinically significant pharmacokinetic interactions [31,37,38], ADs are approved drugs with extensive post-marketing safety data. ADs are widely used across heterogeneous psychiatric populations, including adult and pediatric patients with complex mood and neuropsychiatric disorders, highlighting a favorable safety profile in conditions characterized by profound neurobiological and behavioral alterations [39,40,41]. This clinical familiarity may support a potentially more favorable tolerability framework for combination strategies, although drug–drug interactions remain an important consideration [42]. Potential safety concerns primarily relate to pharmacokinetic interactions, off-target effects at higher AD exposures, and the need to ensure that chemosensitization does not amplify CT-related toxicities; however, available in vivo data indicate that AD–CT combinations have not produced additional systemic toxicity at the doses tested. In this respect, according to the studies included in this review, chemosensitizing effects were achieved at AD concentrations that are within, or below, established therapeutic ranges in humans, without exacerbating systemic toxicity. It can be hypothesized that the use of low-dose ADs may permit chemosensitization while minimizing the risk of clinically relevant drug–drug interactions [43,44,45]. Across the included studies, consistent in vivo findings from syngeneic and xenograft tumor models demonstrate that AD–CT combinations enhance intratumoral drug accumulation, improve antitumor efficacy, and prolong survival without exacerbating systemic toxicity [24,27,28,30]. Collectively, these findings indicate that, although resistance reversal is not exclusive to ADs, selected agents within this class show a reproducible and well-tolerated profile, providing a strong biological and pharmacological rationale for further clinical investigation of ADs as adjunctive agents to overcome multidrug resistance in selected cancer settings.

The variability in AD dosing across studies likely contributes to differences in the extent of P-gp inhibition observed. In vitro experiments used AD concentrations ranging from low to mid micromolar levels, with SSRIs such as FLX and SER generally showing stronger P-gp modulation at lower doses compared to TCA derivatives. In vivo studies also differed in administered doses and treatment schedules, influencing the magnitude of chemosensitization. These discrepancies underline the need for more standardized dosing approaches to better define the minimal effective AD exposure required to modulate MDR mechanisms.

Several limitations should be acknowledged when interpreting the available evidence. First, substantial heterogeneity exists across studies with respect to experimental models, including tumor cell lines, classes of ADs, CT agents, and dosing regimens. This variability limits direct comparison between studies and precludes definitive conclusions regarding the relative efficacy of specific drug combinations. The available evidence is based on a relatively limited number of studies and is largely derived from in vitro and preclinical in vivo models, with a notable lack of controlled clinical trials, thereby restricting immediate clinical extrapolation. Many experimental models relied on artificially selected or genetically engineered multidrug-resistant cell lines, which may not fully recapitulate the complexity and heterogeneity of resistance mechanisms observed in human tumors. Moreover, in vitro and cell culture–based models are inherently limited in their capacity to capture the complexity of drug-related toxicity observed in vivo, as they do not account for pharmacokinetics, organ-specific vulnerability, or cumulative adverse effects.

## 5. Conclusions

ADs can act as effective modulators of multidrug resistance across a range of tumor models, through multiple mechanisms, including both functional inhibition and downregulation of drug efflux transporters, mainly P-gp. This effect does not involve direct cytotoxicity, is dosage-dependent, and is limited to MDR cell lines. These findings support further evaluation of ADs as part of combination strategies to overcome multidrug resistance in cancer. Further studies are needed to expand the available evidence by increasing the number of investigations, further clarifying the biological rationale underlying the selection of AD–CT combinations, and extending current findings to in vivo models and clinical settings in order to evaluate the tolerability and feasibility of these combinations in patients. In addition, future research should focus on identifying the most promising AD-CT pairings based on tumor-specific resistance mechanisms, elucidating dose–response relationships to determine the minimal effective AD exposure required for P-gp modulation, and systematically assessing potential pharmacokinetic interactions. Integrating multi-omics approaches and high-throughput screening may help uncover predictive biomarkers of response and refine patient selection, while innovative AD-based hybrid molecules or delivery systems could further enhance tumor specificity and safety. In parallel, extending investigations to a wider range of tumor types is necessary to clarify whether AD-mediated chemosensitization is cancer-specific or broadly applicable across malignancies. Collectively, these directions will support the translation of AD-augmented chemotherapy into clinical practice and help define its therapeutic value across different oncological contexts

## Data Availability

No new data were created or analyzed in this study. Data sharing is not applicable to this article.

## Supplementary Materials

The following supporting information can be downloaded at: https://www.mdpi.com/article/10.3390/medsci14010126/s1, Section S1: Search strings used for PubMed, Scopus, and PsycInfo; Section S2: Qualitative overview of the included studies; Table S1: Results from the PubMed database search with final decision label; Table S2: Results from the Scopus database search with final decision label; Table S3: Results from the PsycInfo database search with final decision label; Table S4: PRISMA 2020 checklist; Table S5: Risk of Bias assessment of the included in vitro studies using the OHAT RoB tool; Table S6: Full list of references of included studies in chronological order; Figure S1: Risk of Bias assessment of the included in vitro studies using the OHAT RoB tool; Figure S2. Risk of bias assessment of the included animal studies using the SYRCLE’s assessment tool; Table S7. Risk of Bias assessment of the included animal studies using the SYRCLE’s assessment tool.

## Abbreviations

The following abbreviations are used in this manuscript:

ABC	ATP-binding cassette
AD	Antidepressant
BCRP	Breast Cancer Resistance Protein
CT	chemotherapeutic
MDR	Multidrug Resistance
MRP-1	MDR-associated protein 1
OHAT	Office of Health Assessment and Translation
OSF	Open Science Framework
P-gp	P-glycoprotein
RoB	Risk of Bias
SYRCLE	Systematic Review Centre for Laboratory Animal Experimentation
