# Supplementary material for: Combination of Antidepressants and Chemotherapeutic Agents to Overcome P-Glycoprotein-Mediated Resistance in Cancer Patients: A Systematic Review
Source: Med Sci (Basel). 2026 Mar 7;14(1):126. doi: 10.3390/medsci14010126 (PMC13028393; doi:10.3390/medsci14010126)
Supplement: Supplementary file 1 [file medsci-14-00126-s001.zip › medsci-4122593-supplementary.pdf]

Supplementary Material

Index	1
Section S1. Search strings used for PubMed, Scopus, and PsycInfo	1
Table S1. Results from the Pubmed database search with final decision label.	3
Table S2. Results from the Scopus database search with final decision label.	7
Table S3. Results from the PsycInfo database search with final decision label.	24
Table S4. PRISMA 2020 checklist	25
Table S5. Risk of Bias assessment of the included in vitro studies using the OHAT RoB tool	27
Figure S1. Risk of Bias assessment of the included in vitro studies using the OHAT RoB tool	28
Table S6. Full list of references of included studies in chronological order.	28
Figure S2. Risk of bias assessment of the included animal studies using the SYRCLE's assessment tool	30
Table S7. Risk of bias assessment of the included animal studies using the SYRCLE's assessment tool	30
Section S2. Qualitative overview of the included studies	31
References	33

Section S1: Search strings used for Pubmed, Scopus and PsycInfo

The search strings employed were as follows:

a) for PubMed database ("P-glycoprotein"[tiab] OR "P glycoprotein"[tiab] OR "P-gp"[tiab] OR Pgp[tiab] OR PgP[tiab] OR "MDR1"[tiab] OR "ABCB1"[tiab] OR "ABC transporter*"[tiab] OR "drug efflux"[tiab] OR "multidrug resistance protein*"[tiab]) AND ("antidepressant*"[tiab] OR "psychotropic drug*"[tiab] OR "CNS drug*"[tiab] OR "neuropsychiatric drug*"[tiab] OR "selective serotonin reuptake inhibitor*"[tiab] OR SSRI[tiab] OR "serotonin-norepinephrine reuptake inhibitor*"[tiab] OR SNRI[tiab] OR "serotonin antagonist and reuptake inhibitor*"[tiab] OR SARI[tiab] OR "noradrenergic and specific serotonergic antidepressant*"[tiab] OR NaSSA[tiab] OR "tricyclic antidepressant*"[tiab] OR TCA[tiab] OR fluoxetine[tiab] OR paroxetine[tiab] OR sertraline[tiab] OR citalopram[tiab] OR escitalopram[tiab] OR fluvoxamine[tiab] OR vortioxetine[tiab] OR venlafaxine[tiab] OR desvenlafaxine[tiab] OR duloxetine[tiab] OR levomilnacipran[tiab] OR milnacipran[tiab] OR trazodone[tiab] OR nefazodone[tiab] OR mirtazapine[tiab] OR amitriptyline[tiab] OR nortriptyline[tiab] OR imipramine[tiab] OR desipramine[tiab] OR clomipramine[tiab] OR doxepin[tiab] OR trimipramine[tiab] OR protriptyline[tiab] OR "mood disorder*"[tiab] OR "depression treatment"[tiab]) AND ("chemotherapy"[tiab] OR "chemotherapeutic"[tiab] OR "chemotherapeutic agent*"[tiab] OR "anticancer drug*"[tiab] OR "anticancer agent*"[tiab] OR "antineoplastic agent*"[tiab] OR "cytotoxic drug*"[tiab] OR "cancer treatment"[tiab] OR doxorubicin[tiab] OR paclitaxel[tiab] OR cisplatin[tiab] OR carboplatin[tiab] OR vincristine[tiab] OR docetaxel[tiab] OR etoposide[tiab] OR irinotecan[tiab]) AND ("drug resistance"[tiab] OR "multidrug resistance"[tiab] OR resistance[tiab] OR MDR[tiab] OR "reversal of resistance"[tiab] OR "reversal resistance"[tiab] OR revers* OR chemosensitizer*[tiab] OR sensitize[tiab] OR "cross-resistance"[tiab] OR "overcoming resistance"[tiab] OR sensitization[tiab]);

b) for Scopus database (TITLE-ABS-KEY("P-glycoprotein" OR "P glycoprotein" OR "P-gp" OR Pgp OR PgP OR "MDR1" OR "ABCB1" OR "ABC transporter*" OR "drug efflux" OR "multidrug resistance protein*")) AND (TITLE-ABS-KEY("antidepressant*" OR "psychotropic drug*" OR "CNS drug*" OR "neuropsychiatric drug*" OR "selective serotonin reuptake inhibitor*" OR SSRI OR "serotonin-norepinephrine reuptake inhibitor*" OR SNRI OR "serotonin antagonist and reuptake inhibitor*" OR SARI OR "noradrenergic and specific serotonergic antidepressant*" OR NaSSA OR "tricyclic antidepressant*" OR TCA OR fluoxetine OR paroxetine OR sertraline OR citalopram OR escitalopram OR fluvoxamine OR vortioxetine OR venlafaxine OR desvenlafaxine OR duloxetine OR levomilnacipran OR milnacipran

OR trazodone OR nefazodone OR mirtazapine OR amitriptyline OR nortriptyline OR imipramine OR desipramine OR clomipramine OR doxepin OR trimipramine OR protriptyline OR "mood disorder*" OR "depression treatment")) AND (TITLE-ABS-KEY("chemotherapy" OR "chemotherapeutic" OR "chemotherapeutic agent*" OR "anticancer drug*" OR "anticancer agent*"

OR "antineoplastic agent*" OR "cytotoxic drug*" OR "cancer treatment" OR doxorubicin OR paclitaxel OR cisplatin OR carboplatin OR vincristine OR docetaxel OR etoposide OR irinotecan)) AND (TITLE-ABS-KEY("drug resistance" OR "multidrug resistance" OR resistance OR MDR

OR "reversal of resistance" OR "reversal resistance" OR revers* OR chemosensitizer* OR sensitize OR "cross-resistance" OR "overcoming resistance" OR sensitization));

c) for PsycInfo database ("P-glycoprotein" OR "P glycoprotein" OR "P-gp" OR Pgp OR PgP OR MDR1 OR ABCB1 OR "ABC transporter*" OR "drug efflux" OR "multidrug resistance protein*") AND ("antidepressant*" OR "psychotropic drug*" OR "CNS drug*" OR "neuropsychiatric drug*" OR "selective serotonin reuptake inhibitor*" OR SSRI OR "serotonin-norepinephrine reuptake inhibitor*" OR SNRI OR "serotonin antagonist and reuptake inhibitor*" OR SARI OR "noradrenergic and specific serotonergic antidepressant*" OR NaSSA OR "tricyclic antidepressant*" OR TCA OR fluoxetine OR paroxetine OR sertraline OR citalopram OR escitalopram OR fluvoxamine OR vortioxetine OR venlafaxine OR desvenlafaxine OR duloxetine OR levomilnacipran OR milnacipran OR trazodone OR nefazodone OR mirtazapine OR amitriptyline OR nortriptyline OR imipramine OR desipramine OR clomipramine OR doxepin OR trimipramine OR protriptyline OR "mood disorder*" OR "depression treatment") AND ("chemotherapy" OR "chemotherapeutic" OR "chemotherapeutic agent*" OR "anticancer drug*" OR "anticancer agent*" OR "antineoplastic agent*" OR "cytotoxic drug*" OR "cancer treatment" OR doxorubicin OR paclitaxel OR cisplatin OR carboplatin OR vincristine OR docetaxel OR etoposide OR irinotecan OR cancer OR malignant OR malignancy OR neoplasm OR neoplasms OR neoplasia OR neoplastic) AND ("drug resistance" OR "multidrug resistance" OR resistance OR MDR OR "reversal of resistance" OR "reversal resistance" OR revers* OR chemosensitizer* OR sensitize OR "cross-resistance" OR "overcoming resistance" OR sensitization).

Table S1. Results from the Pubmed database search with final decision label.		
	**References**	**Label**
1.	Akamine Y, Yasui-Furukori N, Ieiri I, Uno T. Psychotropic drug-drug interactions involving P-glycoprotein. CNS Drugs. 2012 Nov;26(11):959-73. doi: 10.1007/s40263-012-0008-z. PMID: 23023659.	Review
2.	Pal D, Mitra AK. MDR- and CYP3A4-mediated drug-herbal interactions. Life Sci. 2006 Mar 27;78(18):2131-45. doi: 10.1016/j.lfs.2005.12.010. Epub 2006 Jan 25. PMID: 16442130.	Review
3.	Mannel M. Drug interactions with St John's wort : mechanisms and clinical implications. Drug Saf. 2004;27(11):773-97. doi: 10.2165/00002018-200427110-00003. PMID: 15350151.	Review
4.	Billard C, Merhi F, Bauvois B. Mechanistic insights into the antileukemic activity of hyperforin. Curr Cancer Drug Targets. 2013 Jan;13(1):1-10. PMID: 22924417.	Review
5.	Li Z, Chen C, Chen L, Hu D, Yang X, Zhuo W, Chen Y, Yang J, Zhou Y, Mao M, Zhang X, Xu L, Ju S, Shen J, Wang Q, Dong M, Xie S, Wei Q, Jia Y, Zhou J, Wang L. STAT5a Confers Doxorubicin Resistance to Breast Cancer by Regulating ABCB1. Front Oncol. 2021 Jul 15;11:697950. doi: 10.3389/fonc.2021.697950. Erratum in: Front Oncol. 2022 Mar 09;12:880458. doi: 10.3389/fonc.2022.880458. PMID: 34336684; PMCID: PMC8320598.	Unfocused
6.	Schulz V. Safety of St. John's Wort extract compared to synthetic antidepressants. Phytomedicine. 2006 Feb;13(3):199-204. doi: 10.1016/j.phymed.2005.07.005. Epub 2005 Nov 2. PMID: 16428030.	Review
7.	Liu Y, Hu M. P-glycoprotein and bioavailability-implication of polymorphism. Clin Chem Lab Med. 2000 Sep;38(9):877-81. doi: 10.1515/CCLM.2000.127. PMID: 11097343.	Review
8.	Yu HJ, Xiao GL, Zhao YY, Wang XX, Lan R. Targeting Mitochondrial Metabolism and RNA Polymerase POLRMT to Overcome Multidrug Resistance in Cancer. Front Chem. 2021 Dec 16;9:775226. doi: 10.3389/fchem.2021.775226. PMID: 34976949; PMCID: PMC8716502.	Review
9.	Bin Kanner Y, Teng QX, Ganoth A, Peer D, Wang JQ, Chen ZS, Tsfadia Y. Cytotoxicity and reversal effect of sertraline, fluoxetine, and citalopram on MRP1- and MRP7-mediated MDR. Front Pharmacol. 2023 Nov 2;14:1290255. doi: 10.3389/fphar.2023.1290255. PMID: 38026953; PMCID: PMC10651738.	Unfocused
10.	Drinberg V, Bitcover R, Rajchenbach W, Peer D. Modulating cancer multidrug resistance by sertraline in combination with a nanomedicine. Cancer Lett. 2014 Nov 28;354(2):290-8. doi: 10.1016/j.canlet.2014.08.026. Epub 2014 Aug 27. PMID: 25173796.	Included
11.	Clark H, Knapik LO, Zhang Z, Wu X, Naik MT, Oulhen N, Wessel GM, Brayboy LM. Dysfunctional MDR-1 disrupts mitochondrial homeostasis in the oocyte and ovary. Sci Rep. 2019 Jul 3;9(1):9616. doi: 10.1038/s41598-019-46025-x. PMID: 31270386; PMCID: PMC6610133.	Unfocused
12.	Zhou S, Chan E, Lim LY, Boelsterli UA, Li SC, Wang J, Zhang Q, Huang M, Xu A. Therapeutic drugs that behave as mechanism-based inhibitors of cytochrome P450 3A4. Curr Drug Metab. 2004 Oct;5(5):415-42. doi: 10.2174/1389200043335450. PMID: 15544435.	Review
13.	Kim DG, Bynoe MS. A2A adenosine receptor modulates drug efflux transporter P-glycoprotein at the blood-brain barrier. J Clin Invest. 2016 May 2;126(5):1717-33. doi: 10.1172/JCI76207. Epub 2016 Apr 4. PMID: 27043281; PMCID: PMC4855938.	Unfocused
14.	Shay JW, Homma N, et Al. Abstracts from the 3rd International Genomic Medicine Conference (3rd IGMC 2015) : Jeddah, Kingdom of Saudi Arabia. 30 November - 3 December 2015. BMC Genomics. 2016 Jul 20;17 Suppl 6(Suppl 6):487. doi: 10.1186/s12864-016-2858-0. PMID: 27454254; PMCID: PMC4959372.	Review
15.	Wang JQ, Liu XM, Zhu ZS, Li Z, Xie CZ, Qiao X, Feng YK, Xu JY. Fluoxetine-Conjugated Platinum(IV) Prodrugs Targeting eEF2K and Conquering Multidrug Resistance against	Included

	Triple-Negative Breast Cancer. J Med Chem. 2025 May 8;68(9):9661-9680. doi: 10.1021/acs.jmedchem.5c00352. Epub 2025 Apr 23. PMID: 40265570.	
16.	Zhang H, Bian S, Xu Z, Gao M, Wang H, Zhang J, Zhang M, Ke Y, Wang W, Chen ZS, Xu H. The effect and mechanistic study of encequidar on reversing the resistance of SW620/AD300 cells to doxorubicin. Biochem Pharmacol. 2022 Nov;205:115258. doi: 10.1016/j.bcp.2022.115258. Epub 2022 Sep 27. PMID: 36179932.	Unfocused
17.	Johnson ZL, Chen J. Structural Basis of Substrate Recognition by the Multidrug Resistance Protein MRP1. Cell. 2017 Mar 9;168(6):1075-1085.e9. doi: 10.1016/j.cell.2017.01.041. Epub 2017 Feb 23. PMID: 28238471.	Unfocused
18.	Cheng G, Pi Z, Zhuang X, Zheng Z, Liu S, Liu Z, Song F. The effects and mechanisms of aloe-emodin on reversing adriamycin-induced resistance of MCF-7/ADR cells. Phytother Res. 2021 Jul;35(7):3886-3897. doi: 10.1002/ptr.7096. Epub 2021 Mar 31. PMID: 33792091.	Unfocused
19.	Carson SW, Ousmanou AD, Hoyler SL. Emerging significance of P-glycoprotein in understanding drug disposition and drug interactions in psychopharmacology. Psychopharmacol Bull. 2002 Winter;36(1):67-81. PMID: 12397848.	Review
20.	Tashima T. Brain Cancer Chemotherapy through a Delivery System across the Blood-Brain Barrier into the Brain Based on Receptor-Mediated Transcytosis Using Monoclonal Antibody Conjugates. Biomedicines. 2022 Jul 5;10(7):1597. doi: 10.3390/biomedicines10071597. PMID: 35884906; PMCID: PMC9313144.	Review
21.	Zhang Y, Zhou T, Duan J, Xiao Z, Li G, Xu F. Inhibition of P-glycoprotein and glutathione S-transferase-pi mediated resistance by fluoxetine in MCF-7/ADM cells. Biomed Pharmacother. 2013 Oct;67(8):757-62. doi: 10.1016/j.biopha.2013.04.012. Epub 2013 May 14. PMID: 23731711.	Included
22.	Zong L, Cheng G, Liu S, Pi Z, Liu Z, Song F. Reversal of multidrug resistance in breast cancer cells by a combination of ursolic acid with doxorubicin. J Pharm Biomed Anal. 2019 Feb 20;165:268-275. doi: 10.1016/j.jpba.2018.11.057. Epub 2018 Dec 11. PMID: 30572191.	Unfocused
23.	Pajeva IK, Wiese M, Cordes HP, Seydel JK. Membrane interactions of some catamphiphilic drugs and relation to their multidrug-resistance-reversing ability. J Cancer Res Clin Oncol. 1996;122(1):27-40. doi: 10.1007/BF01203070. PMID: 8543589; PMCID: PMC12201749.	Unfocused
24.	Ho YC, Chiu WC, Chen JY, Huang YH, Teng YN. Reversal potentials of Tween 20 in ABC transporter-mediated multidrug-resistant cancer and treatment-resistant depression through interacting with both drug-binding and ATP-binding areas on MDR proteins. J Drug Target. 2025 Mar;33(3):410-423. doi: 10.1080/1061186X.2024.2429006. Epub 2024 Nov 18. PMID: 39530732.	Unfocused
25.	Yang C, Yuan H, Gu J, Xu D, Wang M, Qiao J, Yang X, Zhang J, Yao M, Gu J, Tu H, Gan Y. ABCA8-mediated efflux of taurocholic acid contributes to gemcitabine insensitivity in human pancreatic cancer via the S1PR2-ERK pathway. Cell Death Discov. 2021 Jan 11;7(1):6. doi: 10.1038/s41420-020-00390-z. PMID: 33431858; PMCID: PMC7801517.	Unfocused
26.	Duarte D, Nunes M, Ricardo S, Vale N. Combination of Antimalarial and CNS Drugs with Antineoplastic Agents in MCF-7 Breast and HT-29 Colon Cancer Cells: Biosafety Evaluation and Mechanism of Action. Biomolecules. 2022 Oct 16;12(10):1490. doi: 10.3390/biom12101490. PMID: 36291699; PMCID: PMC9599492.	Included
27.	Sarginson JE, Lazzeroni LC, Ryan HS, Ershoff BD, Schadberg AF, Murphy GM Jr. ABCB1 (MDR1) polymorphisms and antidepressant response in geriatric depression. Pharmacogenet Genomics. 2010 Aug;20(8):467-75. doi: 10.1097/FPC.0b013e32833b593a. PMID: 20555295.	Unfocused
28.	Jaffrézou JP, Chen KG, Durán GE, Muller C, Bordier C, Laurent G, Sikic BI, Levade T. Inhibition of lysosomal acid sphingomyelinase by agents which reverse multidrug resistance. Biochim Biophys Acta. 1995 Apr 6;1266(1):1-8. doi: 10.1016/0167-4889(94)00219-5. PMID: 7718613.	Unfocused

29.	Walter RD, Seth M, Bhaduri AP. Reversal of chloroquine resistance in Plasmodium falciparum by CDR 87/209 and analogues. Trop Med Parasitol. 1993 Mar;44(1):5-8. PMID: 8516635.	Unfocused
30.	Palmeira A, Rodrigues F, Sousa E, Pinto M, Vasconcelos MH, Fernandes MX. New uses for old drugs: pharmacophore-based screening for the discovery of P-glycoprotein inhibitors. Chem Biol Drug Des. 2011 Jul;78(1):57-72. doi: 10.1111/j.1747-0285.2011.01089.x. Epub 2011 Mar 29. PMID: 21235729.	Included
31.	Peer D, Dekel Y, Melikhov D, Margalit R. Fluoxetine inhibits multidrug resistance extrusion pumps and enhances responses to chemotherapy in syngeneic and in human xenograft mouse tumor models. Cancer Res. 2004 Oct 15;64(20):7562-9. doi: 10.1158/0008-5472.CAN-03-4046. PMID: 15492283.	Included
32.	Argov M, Kashi R, Peer D, Margalit R. Treatment of resistant human colon cancer xenografts by a fluoxetine-doxorubicin combination enhances therapeutic responses comparable to an aggressive bevacizumab regimen. Cancer Lett. 2009 Feb 8;274(1):118-25. doi: 10.1016/j.canlet.2008.09.005. Epub 2008 Oct 11. PMID: 18851896.	Included
33.	Shi J, Pabon K, Ding R, Scotto KW. ABCG2 and SLC1A5 functionally interact to rewire metabolism and confer a survival advantage to cancer cells under oxidative stress. J Biol Chem. 2024 Jun;300(6):107299. doi: 10.1016/j.jbc.2024.107299. Epub 2024 Apr 18. PMID: 38641063; PMCID: PMC11131071.	Unfocused
34.	Feng S, Zhou H, Wu D, Zheng D, Qu B, Liu R, Zhang C, Li Z, Xie Y, Luo HB. Nobiletin and its derivatives overcome multidrug resistance (MDR) in cancer: total synthesis and discovery of potent MDR reversal agents. Acta Pharm Sin B. 2020 Feb;10(2):327-343. doi: 10.1016/j.apsb.2019.07.007. Epub 2019 Jul 31. PMID: 32082977; PMCID: PMC7016283.	Unfocused
35.	Barbieri F, Alama A, Tasso B, Boido V, Bruzzo C, Sparatore F. Quinolizidinyl derivatives of iminodibenzyl and phenothiazine as multidrug resistance modulators in ovarian cancer cells. Invest New Drugs. 2003 Nov;21(4):413-20. doi: 10.1023/a:1026295017158. PMID: 14586208.	Unfocused
36.	Balayssac D, Cayre A, Authier N, Ling B, Maublant J, Eschalier A, Penault-Llorca F, Coudore F. Involvement of the multidrug resistance transporters in cisplatin-induced neuropathy in rats. Comparison with the chronic constriction injury model and monoarthritic rats. Eur J Pharmacol. 2006 Aug 21;544(1-3):49-57. doi: 10.1016/j.ejphar.2006.06.055. Epub 2006 Jun 29. PMID: 16859677.	Unfocused
37.	Su JL, Wang CH, Kang HG, Zhang J, Wang BZ, Liu MR, Zhao J, Liu L. Association between MDR1 gene of gastrointestinal tumors, the expression of P-glycoprotein and resistance to chemotherapeutic drugs. Oncol Lett. 2017 Sep;14(3):3510-3514. doi: 10.3892/ol.2017.6642. Epub 2017 Jul 20. PMID: 28927106; PMCID: PMC5588068.	Unfocused
38.	Özkaya Gül S, Şimşek B, Yıldız F, Aydemir E. Cytotoxic Effect of Escitalopram/Etoposide Combination on Etoposide-Resistant Lung Cancer. Pharmaceuticals (Basel). 2025 Apr 5;18(4):531. doi: 10.3390/ph18040531. PMID: 40283966; PMCID: PMC12030030.	Included
39.	Altinoz MA, Gedikoglu G, Sav A, Ozcan E, Ozdilli K, Bilir A, Del Maestro RF. Medroxyprogesterone acetate induces c6 glioma chemosensitization via antidepressant-like lysosomal phospholipidosis/myelinosis in vitro. Int J Neurosci. 2007 Oct;117(10):1465-80. doi: 10.1080/00207450701540062. PMID: 17729157.	Unfocused
40.	Jendželovská Z, Jendželovský R, Hiľovská L, Kovaľ J, Mikeš J, Fedoročko P. Single pre-treatment with hypericin, a St. John's wort secondary metabolite, attenuates cisplatin- and mitoxantrone-induced cell death in A2780, A2780cis and HL-60 cells. Toxicol In Vitro. 2014 Oct;28(7):1259-73. doi: 10.1016/j.tiv.2014.06.011. Epub 2014 Jun 30. PMID: 24994473.	Unfocused
41.	Sampson A, Peterson BG, Tan KW, Iram SH. Doxorubicin as a fluorescent reporter identifies novel MRP1 (ABCC1) inhibitors missed by calcein-based high content screening	Unfocused

	of anticancer agents. Biomed Pharmacother. 2019 Oct;118:109289. doi: 10.1016/j.biopha.2019.109289. Epub 2019 Aug 8. PMID: 31401398.	
42.	Panchagnula R, Bansal T, Varma MV, Kaul CL. Co-treatment with grapefruit juice inhibits while chronic administration activates intestinal P-glycoprotein-mediated drug efflux. Pharmazie. 2005 Dec;60(12):922-7. PMID: 16398269.	Unfocused
43.	Di Nicolantonio F, Knight LA, Glaysher S, Whitehouse PA, Mercer SJ, Sharma S, Mills L, Prin A, Johnson P, Charlton PA, Norris D, Cree IA. Ex vivo reversal of chemoresistance by tariquidar (XR9576). Anticancer Drugs. 2004 Oct;15(9):861-9. doi: 10.1097/00001813-200410000-00006. PMID: 15457126.	Unfocused
44.	Fan D, Poste G, Obrian C, Seid C, Ward N, Earnest L, Fidler I. Chemosensitization of murine fibrosarcoma cells to drugs affected by the multidrug resistance phenotype by the antidepressant trazodone - an experimental-model for the reversal of intrinsic drug-resistance. Int J Oncol. 1992 Dec;1(7):735-42. PMID: 21584609.	Included
45.	Gao GL, Wan HY, Zou XS, Chen WX, Chen YQ, Huang XZ. [Relationship between the expression of P-glycoprotein, glutathione S-transferase-pi and thymidylate synthase proteins and adenosine triphosphate tumor chemosensitivity assay in cervical cancer]. Zhonghua Fu Chan Ke Za Zhi. 2007 Mar;42(3):201-5. Chinese. PMID: 17537309.	Unfocused
46.	Zhou CZ, Li Y, Xu J. [Correlation between p53 gene mutation and the expression of tumor drug resistance genes in lung cancer and its clinical significance]. Zhonghua Jie He He Hu Xi Za Zhi. 2004 Oct;27(10):678-82. Chinese. PMID: 16200870.	Unfocused

Table S2. Results from the Scopus database search with final decision label.		
1.	Wang Y. Ligand supplementation restores the cancer therapy efficacy of the antirheumatic drug auranofin from serum inactivation. Nature communications. 12.2025;16(1). doi: 10.1038/s41467-025-62634-9	Unfocused
2.	Wang J. Fluoxetine-conjugated platinum(IV) prodrugs targeting eEF2K and conquering multidrug resistance against triple-negative breast cancer. Journal of Medicinal Chemistry. 05.2025;68(9):9661-9680. doi: 10.1021/acs.jmedchem.5c00352.	Duplicated
3.	Hildebrandt J. In vivo and in vitro pharmacokinetic studies of a dual topoisomerase I/II inhibitor. ACS Pharmacology & Translational Science. 04.2025;8(4):1050-1071. doi: 10.1021/acsptsci.4c00596.	Unfocused
4.	Özkaya Gül S. Cytotoxic effect of Escitalopram/Etoposide combination on etoposide-resistant lung cancer. Pharmaceuticals (Basel, Swiderland). 04.2025;18(4):531. doi: 10.3390/ph18040531.	Duplicated
5.	Grigoreva TA. PROTAC-attractive site as a new target for suppressing P-glycoprotein activity. Archives of biochemistry and biophysics. 02.2025;764:110258. doi: 10.1016/j.abb.2024.110258.	Unfocused
6.	Ho Y. Reversal potentials of tween 20 in ABC transporter-mediated multidrug-resistant cancer and treatment-resistant depression through interacting with both drug-binding and ATP-binding areas on MDR proteins. Journal of Drug Targeting. 03.2025;33(3):410-423. doi: 10.1080/1061186x.2024.2429006.	Duplicated
7.	Liang F. Integrated PBPK-EO modeling of osimertinib to predict plasma concentrations and intracranial EGFR engagement in patients with brain metastases. Scientific reports. 12.2024;14(1). doi: 10.1038/s41598-024-63743-z.	Unfocused
8.	Expert consensus on the clinical application of oral small-molecule antiviral drugs against COVID-19. Infectious Diseases & Immunity. 2024;4(4):158-169. doi: 10.1097/id9.0000000000000139.	Review
9.	Su Y. Graphene quantum dots eradicate resistant and metastatic cancer cells by enhanced interfacial inhibition. Advanced healthcare materials. 07.2024;13(19). doi: 10.1002/adhm.202304648.	Unfocused
10.	Amaroli A. The bright side of curcumin: A narrative Review of its therapeutic potential in cancer management. Cancers. 07.2024;16(14):2580. doi: 10.3390/cancers16142580.	Review
11.	Lennep BW. Enzalutamide: Understanding and managing drug interactions to improve patient safety and drug efficacy. Drug safety. 07.2024;47(7):617-641. doi: 10.1007/s40264-024-01415-7.	Review
12.	Shi J. ABCG2 and SLC1A5 functionally interact to rewire metabolism and confer a survival advantage to cancer cells under oxidative stress. The Journal of biological chemistry. 06.2024;300(6):107299. doi: 10.1016/j.jbc.2024.107299.	Unfocused
13.	Grullon JR. Do P-glycoprotein medications alter the risk of ventriculoperitoneal shunt in adults with hydrocephalus? Journal of clinical pharmacology. 03.2024;64(3):371-377. doi: 10.1002/jcph.2359.	Unfocused
14.	Malik JR. Chemotherapy in pediatric brain tumor and the challenge of the blood–brain barrier. Cancer medicine (Malden, MA). 12.2023;12(23):21075-21096. doi: 10.1002/cam4.6647.	Review
15.	Langarizadeh MA. The trimethoxyphenyl (TMP) functional group: A versatile pharmacophore. Medicinal chemistry research. 12.2023;32(12):2473-2500. doi: 10.1007/s00044-023-03153-4.	Review
16.	Stöllberger C. Cannabidiol's impact on drug-metabolization. European journal of internal medicine 12.2023;118:6-13.	Review

17.	Fu T. The important role of transporter structures in drug disposition, efficacy, and toxicity. Drug Metabolism and Disposition. 10.2023;51(10):1316-1323. doi: 10.1124/dmd.123.001275.	Review
18.	Machkouri C. Oral anticancer agents as generators of relevant pharmacokinetic interactions. Bulletin du Cancer. 06.2023;110(6):676-684. doi: 10.1016/j.bulcan.2023.04.016.	Review
19.	Kumar V. Drug delivery and testing via 3D printing. Bioprinting (Amsterdam, Netherlands). 12.2023;36:e00298. doi: 10.1016/j.bprint.2023.e00298.	Review
20.	Schäfer J. In silico and in vitro identification of P-glycoprotein inhibitors from a library of 375 phytochemicals. International journal of molecular sciences. 06.2023;24(12):10240. doi: 10.3390/ijms241210240.	Unfocused
21.	Gomez-Zepeda D. Functional and targeted proteomics characterization of a human primary endothelial cell model of the blood-brain barrier (BBB) for drug permeability studies. Toxicology and applied pharmacology. 04.2023;465:116456. doi: 10.1016/j.taap.2023.116456.	Unfocused
22.	Babayeva M. Cannabis pharmacogenomics: A path to personalized medicine. Current issues in molecular biology. 04.2023;45(4):3479-3514. doi: 10.3390/cimb45040228.	Review
23.	Roundhill EA. Exploiting the stemness and chemoresistance transcriptome of ewing sarcoma to identify candidate therapeutic targets and drug-repurposing candidates. Cancers. 02.2023;15(3):769. doi: 10.3390/cancers15030769.	Unfocuse
24.	Bin Kanner Y, Teng QX, Ganoth A, Peer D, Wang JQ, Chen ZS, Tsfadia Y. Cytotoxicity and reversal effect of sertraline, fluoxetine, and citalopram on MRP1- and MRP7-mediated MDR. Front Pharmacol. 2023 Nov 2;14:1290255. doi: 10.3389/fphar.2023.1290255. PMID: 38026953; PMCID: PMC10651738.	Duplicated
25.	Czigle S. Pharmacokinetic and pharmacodynamic herb-drug interactions—part I. herbal medicines of the central nervous system. PeerJ (San Francisco, CA). 11.2023;11:e16149. doi: 10.7717/peerj.16149.	Review
26.	Chetri S. The culmination of multidrug-resistant efflux pumps vs. meager antibiotic arsenal era: Urgent need for an improved new generation of EPIs. Frontiers in microbiology. 04.2023;14. doi: 10.3389/fmicb.2023.1149418.	Review
27.	Zhang T. An in vitro human mammary epithelial cell permeability assay to assess drug secretion into breast milk. International journal of pharmaceutics: X. 12.2022;4:100122. doi: 10.1016/j.ijpx.2022.100122.	Unfocused
28.	Zhang H, Bian S, Xu Z, Gao M, Wang H, Zhang J, Zhang M, Ke Y, Wang W, Chen ZS, Xu H. The effect and mechanistic study of encequidar on reversing the resistance of SW620/AD300 cells to doxorubicin. Biochem Pharmacol. 2022 Nov;205:115258. doi: 10.1016/j.bcp.2022.115258. Epub 2022 Sep 27. PMID: 36179932.	Unfocused
29.	Duarte D. Combination of antimalarial and CNS drugs with antineoplastic agents in MCF-7 breast and HT-29 colon cancer cells: Biosafety evaluation and mechanism of action. Biomolecules (Basel, Swiderland). 10.2022;12(10):1490. doi: 10.3390/biom12101490.	Duplicated
30.	Verma A. Molecular mechanisms regulating the pharmacological actions of icariin with special focus on PI3K-AKT and nrf-2 signaling pathways. Molecular biology reports. 09.2022;49(9):9023-9032. doi: 10.1007/s11033-022-07778-3.	Review
31.	Caetano-Pinto P. In vitro characterization of renal drug transporter activity in kidney cancer. International journal of molecular sciences. 09.2022;23(17):10177. doi: 10.3390/ijms231710177.	Unfocused
32.	Eng ME. ATP-binding cassette (ABC) drug transporters in the developing blood–brain barrier: Role in fetal brain protection. Cellular and molecular life sciences : CMLS. 08.2022;79(8). doi: 10.1007/s00018-022-04432-w.	Review

33.	Tashima T. Brain cancer chemotherapy through a delivery system across the blood-brain barrier into the brain based on receptor-mediated transcytosis using monoclonal antibody conjugates. Biomedicines. 07.2022;10(7):1597. doi: 10.3390/biomedicines10071597.	Review
34.	Rekha U V. Review on anticancer properties of piperine in oral cancer: Therapeutic perspectives. Research journal of pharmacy and technology. 07.2022;15(7):3338-3342. doi: 10.52711/0974-360x.2022.00558.	Review
35.	Lu R. Strategies and mechanism in reversing intestinal drug efflux in oral drug delivery. Pharmaceutics. 06.2022;14(6):1131. doi: 10.3390/pharmaceutics14061131.	Review
36.	Tron C. A robust and Fast/Multiplex pharmacogenetics assay to simultaneously analyze 17 clinically relevant genetic polymorphisms in CYP3A4, CYP3A5, CYP1A2, CYP2C9, CYP2C19, CYP2D6, ABCB1, and VKORC1 genes. Pharmaceuticals (Basel, Swiderland). 05.2022;15(5):637. doi: 10.3390/ph15050637.	Unfocused
37.	Nwabufo CK. Relevance of ABC transporters in drug development. Current Drug Metabolism. 05.2022;23(6):434-446. doi: 10.2174/1389200223666220621113524.	Review
38.	Järvinen E. The role of uptake and efflux transporters in the disposition of glucuronide and sulfate conjugates. Frontiers in pharmacology. 01.2022;12. doi: 10.3389/fphar.2021.802539.	Review
39.	Volpe DA. Do differences in cell lines and methods used for calculation of IC50 values influence categorisation of drugs as P-glycoprotein substrates and inhibitors? Xenobiotica. 07.2022;52(7):751-757. doi: 10.1080/00498254.2022.2135040.	Unfocused
40.	Martins V. A commentary on the use of pharmacoenhancers in the pharmaceutical industry and the implication for DMPK drug discovery strategies. Xenobiotica. 08.2022;52(8):786-796. doi: 10.1080/00498254.2022.2130838.	Review
41.	Yu H. Targeting mitochondrial metabolism and RNA polymerase POLRMT to overcome multidrug resistance in cancer. Frontiers in chemistry. 12.2021;9. doi: 10.3389/fchem.2021.775226.	Review
42.	Tymon-Rosario J. Microtubule-interfering drugs: Current and future roles in epithelial ovarian cancer treatment. Cancers. 12.2021;13(24):6239. doi: 10.3390/cancers13246239.	Review
43.	Maeda K. Classification of drugs for evaluating drug interaction in drug development and clinical management. Drug metabolism and pharmacokinetics. 12.2021;41:100414. doi: 10.1016/j.dmpk.2021.100414.	Review
44.	Chen X. Tetrahydrocannabinol and its major metabolites are not (or are poor) substrates or inhibitors of human P-glycoprotein [ATP-binding cassette (ABC) B1] and breast cancer resistance protein (ABCG2). Drug Metabolism and Disposition. 10.2021;49(10):910-918. doi: 10.1124/dmd.121.000505.	Unfocused
45.	Pilla Reddy V. Food constituent– and herb–drug interactions in oncology: Influence of quantitative modelling on drug labelling. British journal of clinical pharmacology. 10.2021;87(10):3988-4000. doi: 10.1111/bcp.14822.	Unfocused
46.	Wang M. Abnormal saccharides affecting cancer multi-drug resistance (MDR) and the reversal strategies. European journal of medicinal chemistry. 08.2021;220:113487. doi: 10.1016/j.ejmech.2021.113487.	Review
47.	Niedrig DF. Clinical relevance of a 16-gene pharmacogenetic panel test for medication management in a cohort of 135 patients. Journal of clinical medicine. 08.2021;10(15):3200. doi: 10.3390/jcm10153200.	Unfocused
48.	Li Z. STAT5a confers doxorubicin resistance to breast cancer by regulating ABCB1. Frontiers in oncology. 07.2021;11. doi: 10.3389/fonc.2021.697950.	Unfocused
49.	Sato S. Translational CNS steady-state drug disposition model in rats, monkeys, and humans for quantitative prediction of brain-to-plasma and cerebrospinal fluid-to-plasma unbound concentration ratios. The AAPS journal. 07.2021;23(4). doi: 10.1208/s12248-021-00609-6.	Unfocused

50.	Cheng G, Pi Z, Zhuang X, Zheng Z, Liu S, Liu Z, Song F. The effects and mechanisms of aloe-emodin on reversing adriamycin-induced resistance of MCF-7/ADR cells. Phytother Res. 2021 Jul;35(7):3886-3897. doi: 10.1002/ptr.7096. Epub 2021 Mar 31. PMID: 33792091.	Duplicated
51.	Yang C. ABCA8-mediated efflux of taurocholic acid contributes to gemcitabine insensitivity in human pancreatic cancer via the S1PR2-ERK pathway. Cell death discovery. 06.2021;7(1). doi: 10.1038/s41420-020-00390-z.	Unfocused
52.	Choi M. Pharmacokinetic Drug–Drug interactions and Herb–Drug interactions. Pharmaceutics. 05.2021;13(5):610. doi: 10.3390/pharmaceutics13050610.	Editorial
53.	Zhang H. Thirteen bisbenzylisoquinoline alkaloids in five chinese medicinal plants: Botany, traditional uses, phytochemistry, pharmacokinetic and toxicity studies. Journal of ethnopharmacology. 03.2021;268:113566. doi: 10.1016/j.jep.2020.113566.	Review
54.	Bruckmueller H. ABCB1, ABCG2, ABCC1, ABCC2, and ABCC3 drug transporter polymorphisms and their impact on drug bioavailability: What is our current understanding? Expert Opinion on Drug Metabolism & Toxicology. 04.2021;17(4):369-396. doi: 10.1080/17425255.2021.1876661.	Review
55.	Kucwaj-Brysz K. The relationship between stereochemical and both, pharmacological and ADME-tox, properties of the potent hydantoin 5-HT7R antagonist MF-8. Bioorganic chemistry. 01.2021;106:104466. doi: 10.1016/j.bioorg.2020.104466.	Unfocused
56.	Sasabe H. In vitro evaluations for pharmacokinetic drug-drug interactions of a novel serotonin-dopamine activity modulator, brexpiprazole. Xenobiotica. 05.2021;51(5):522-535. doi: 10.1080/00498254.2021.1897898.	Unfocused
57.	Bechtold B. Multi-factorial pharmacokinetic interactions: Unraveling complexities in precision drug therapy. Expert Opinion on Drug Metabolism & Toxicology. 04.2021;17(4):397-412. doi: 10.1080/17425255.2021.1867105.	Review
58.	Bhoopathy S. Methods in molecular biology enzyme kinetics in drug metabolism. Principles and Experimental Considerations for In Vitro Transporter Interaction Assays. 2021:339-365. doi: 10.1007/978-1-0716-1554-6_13.	Review
59.	Carvalho Henriques B. How can drug metabolism and transporter genetics inform psychotropic prescribing? Frontiers in genetics. 12.2020;11. doi: 10.3389/fgene.2020.491895.	Review
60.	Lai J. Clinical perspective of FDA approved drugs with P-glycoprotein inhibition activities for potential cancer therapeutics. Frontiers in oncology. 11.2020;10. doi: 10.3389/fonc.2020.561936.	Review
61.	Roncato R. CDK4/6 inhibitors in breast cancer treatment: Potential interactions with drug, gene, and pathophysiological conditions. International journal of molecular sciences. 09.2020;21(17):6350. doi: 10.3390/ijms21176350.	Review
62.	Wanat K. Biological barriers, and the influence of protein binding on the passage of drugs across them. Molecular biology reports. 04.2020;47(4):3221-3231. doi: 10.1007/s11033-020-05361-2.	Review
63.	Zhou Y. Pharmacogenomic network analysis of the gene-drug interaction landscape underlying drug disposition. Computational and structural biotechnology journal. 2020;18:52-58. doi: 10.1016/j.csbj.2019.11.010.	Unfocused
64.	Yang W. BATF2 inhibits chemotherapy resistance by suppressing AP-1 in vincristine-resistant gastric cancer cells. Cancer chemotherapy and pharmacology. 12.2019;84(6):1279-1288. doi: 10.1007/s00280-019-03958-4.	Unfocused
65.	Sampson A. Doxorubicin as a fluorescent reporter identifies novel MRP1 (ABCC1) inhibitors missed by calcein-based high content screening of anticancer agents. Biomedicine & pharmacotherapy. 10.2019;118:109289. doi: 10.1016/j.biopha.2019.109289.	Unfocused
66.	Kadioglu O. A machine learning-based prediction platform for P-glycoprotein modulators and its validation by molecular docking. Cells (Basel, Swiderland). 10.2019;8(10):1286. doi: 10.3390/cells8101286.	Unfocused

67.	Mirzaev KB. ADME pharmacogenetics: Future outlook for russia. Pharmacogenomics. 07.2019;20(11):847-865. doi: 10.2217/pgs-2019-0013.	Review
68.	Bahmad HF. Cancer stem cells in neuroblastoma: Expanding the therapeutic frontier. Frontiers in molecular neuroscience. 05.2019;12. doi: 10.3389/fnmol.2019.00131	Review
69.	Zong L. Reversal of multidrug resistance in breast cancer cells by a combination of ursolic acid with doxorubicin. Journal of pharmaceutical and biomedical analysis. 02.2019;165:268-275. doi: 10.1016/j.jpba.2018.11.057.	Unfocused
70.	Villanueva S. ABC transporters in extrahepatic tissues: Pharmacological regulation in heart and intestine. Current Medicinal Chemistry. 05.2019;26(7):1155-1184. doi: 10.2174/0929867325666180327092639	Review
71.	Ekinci E. Repurposing disulfiram as an anti-cancer agent: Updated Review on literature and patents. Recent Patents on Anti-Cancer Drug Discovery. 08.2019;14(2):113-132. doi: 10.2174/1574892814666190514104035.	Review
72.	Liu L. Advances in experimental medicine and biology drug transporters in drug disposition, effects and toxicity. Contributions of Drug Transporters to Blood-Brain Barriers. 2019:407-466. doi: 10.1007/978-981-13-7647-4_9.	Book Chapter
73.	Bossennec M. MDR1 in immunity: Friend or foe? OncoImmunology. 12.2018;7(12):e1499388. doi: 10.1080/2162402x.2018.1499388.	Review
74.	Soleymani S. Clinical risks of st John's wort (hypericum perforatum) co-administration. Expert Opinion on Drug Metabolism & Toxicology. 10.2017;13(10):1047-1062. doi: 10.1080/17425255.2017.1378342.	Review
75.	Bahramsoltani R. Pharmacokinetic interactions of curcuminoids with conventional drugs: A Review. Journal of ethnopharmacology. 09.2017;209:1-12. doi: 10.1016/j.jep.2017.07.022.	Review
76.	Liu Y. Fluoxetine enhances cellular chemosensitivity to cisplatin in cervical cancer. International Journal of Clinical and Experimental Medicine. 2017;10(7):10521-10527.	Included
77.	Ellis CT. Inferior survival rates after chemoradiation for rectal cancer without Surgery—Reply. JAMA oncology. 06.2017;3(6):859. doi: 10.1001/jamaoncol.2017.0407.	Editorial
78.	Johnson ZL. Structural basis of substrate recognition by the multidrug resistance protein MRP1. Cell. 03.2017;168(6):1075-1085.e9. doi: 10.1016/j.cell.2017.01.041.	Unfocused
79.	Su J. Association between MDR1 gene of gastrointestinal tumors, the expression of P-glycoprotein and resistance to chemotherapeutic drugs. Oncology letters. 09.2017;14(3):3510-3514. doi: 10.3892/ol.2017.6642.	Unfocused
80.	McCormick A. In vitro assessment of the roles of drug transporters in the disposition and drug–drug interaction potential of olaparib. Xenobiotica. 10.2017;47(10):903-915. doi: 10.1080/00498254.2016.1241449.	Unfocused
81.	A. Marcath L. Comprehensive assessment of cytochromes P450 and transporter genetics with endoxifen concentration during tamoxifen treatment. Pharmacogenetics and Genomics. 2017;27(11):402-409. doi: 10.1097/fpc.0000000000000311.	Unfocused
82.	Imenshahidi M. Berberis vulgarisand berberine: An update Review. Phytotherapy research. 11.2016;30(11):1745-1764. doi: 10.1002/ptr.5693.	Review
83.	Jendželovská Z. Hypericin in the light and in the dark: Two sides of the same coin. Frontiers in plant science. 05.2016;7(may2016). doi: 10.3389/fpls.2016.00560.	Review
84.	Kim D. A2A adenosine receptor modulates drug efflux transporter P-glycoprotein at the blood-brain barrier. The Journal of clinical investigation. 05.2016;126(5):1717-1733. doi: 10.1172/jci76207.	Unfocused
85.	Burt T. Microdosing and other phase 0 clinical trials: Facilitating translation in drug development. Clinical and translational science. 04.2016;9(2):74-88. doi: 10.1111/cts.12390.	Review
86.	Saaby L. IPEC-J2 MDR1, a novel high-resistance cell line with functional expression of human P-glycoprotein (ABCB1) for drug screening studies. Molecular Pharmaceutics. 02.2016;13(2):640-652. doi: 10.1021/acs.molpharmaceut.5b00874.	Unfocused

87.	Takenaka T. Application of a human intestinal epithelial cell monolayer to the prediction of oral drug absorption in humans as a superior alternative to the caco-2 cell monolayer. Journal of pharmaceutical sciences. 02.2016;105(2):915-924. doi: 10.1016/j.xphs.2015.11.035.	Unfocused
88.	Bosilkovska M. Severe vincristine-induced neuropathic pain in a CYP3A5 nonexpressor with reduced CYP3A4/5 activity: Case study. Clinical therapeutics. 01.2016;38(1):216-220. doi: 10.1016/j.clinthera.2015.10.017	Case
89.	Mora E. Vincristine-induced peripheral neuropathy in pediatric cancer patients. American Journal of Cancer Research. 2016;6(11):2416-2430.	Review
90.	Ito N. Prediction of drug transfer into milk considering breast cancer resistance protein (BCRP)-mediated transport. Pharmaceutical research. 02.2015;32(8):2527-2537. doi: 10.1007/s11095-015-1641-2.	Unfocused
91.	Wolking S. Impact of genetic polymorphisms of ABCB1 (MDR1, P-glycoprotein) on drug disposition and potential clinical implications: Update of the literature. Clinical pharmacokinetics. 07.2015;54(7):709-735. doi: 10.1007/s40262-015-0267-1.	Review
92.	Li W. MDR1 will play a key role in pharmacokinetic changes under hypoxia at high altitude and its potential regulatory networks. Drug Metabolism Reviews. 04.2015;47(2):191-198. doi: 10.3109/03602532.2015.1007012.	Review
93.	Wang, Z. Y., Chen, M., Zhu, L. L., Yu, L. S., Zeng, S., Xiang, M. X., & Zhou, Q. (2015). Pharmacokinetic drug interactions with clopidogrel: updated Review and risk management in combination therapy. Therapeutics and clinical risk management, 449-467. 10.2147/TCRM.S80437	Review
94.	Martin E. Rationale and design of a randomized double-blind clinical trial in breast cancer: Dextromethorphan in chemotherapy-induced peripheral neuropathy. Contemporary clinical trials. 03.2015;41:146-151. doi: 10.1016/j.cct.2015.01.012.	Unfocused
95.	Gupta P. Polymeric drug-delivery systems: Role in P-gp efflux system inhibition. Critical Reviews in Therapeutic Drug Carrier Systems. 2015;32(3):247-275. doi: 10.1615/critrevtherdrugcarriersyst.2015011592	Review
96.	Li X. The challenge of efflux-mediated antibiotic resistance in gram-negative bacteria. Clinical microbiology Reviews. 04.2015;28(2):337-418. doi: 10.1128/cmr.00117-14.	Review
97.	Akilandeswari, K., & Ruckmani, K. (2015). Studies on anti microbial potential of non-antibiotics on resistant bacteria-A Review. Journal of Young Pharmacists, 7(2), 63. DOI: 10.5530/jyp.2015.2.2	Review
98.	Drinberg, V., Bitcover, R., Rajchenbach, W., & Peer, D. (2014). Modulating cancer multidrug resistance by sertraline in combination with a nanomedicine. Cancer letters, 354(2), 290-298. 10.1016/j.canlet.2014.08.026	Unfocused
99.	Pahnke, J., Fröhlich, C., Paarmann, K., Krohn, M., Bogdanovic, N., Årsland, D., & Winblad, B. (2014). Cerebral ABC transporter-common mechanisms may modulate neurodegenerative diseases and depression in elderly subjects. Archives of medical research, 45(8), 738-743. 10.1016/j.arcmed.2014.10.010	Review
100.	Saeed, M., Zeino, M., Kadioglu, O., Volm, M., & Efferth, T. (2014). Overcoming of P-glycoprotein-mediated multidrug resistance of tumors in vivo by drug combinations. Synergy, 1(1), 44-58. 10.1016/j.synres.2014.07.002	Review
101.	JIA, M., CHEN, Z., SHI, L., ZHAO, B., WU, X., & WU, Y. (2014). Key protein expressions in the insulin-like growth factor-1 signal pathway involved in the resistance of ovarian cancer to cisplatin. Chinese Journal of Clinical Oncology, 286-290. 10.3969/j.issn.1000-8179.20131238	Unfocused
102.	Jiang, P., Mukthavavam, R., Chao, Y., Bharati, I. S., Fogal, V., Pastorino, S., ... & Kesari, S. (2014). Novel anti-glioblastoma agents and therapeutic combinations identified from a collection of FDA approved drugs. Journal of translational medicine, 12(1), 13. 10.1186/1479-5876-12-13	Unfocused

103.	Chaves, C., Shawahna, R., Jacob, A., Scherrmann, J. M., & Declèves, X. (2014). Human ABC transporters at blood-CNS interfaces as determinants of CNS drug penetration. Current Pharmaceutical Design, 20(10), 1450-1462.	Review
104.	Jendželovská Z, Jendželovský R, Hiľovská L, Kovaľ J, Mikeš J, Fedoročko P. Single pre-treatment with hypericin, a St. John's wort secondary metabolite, attenuates cisplatin- and mitoxantrone-induced cell death in A2780, A2780cis and HL-60 cells. Toxicol In Vitro. 2014 Oct;28(7):1259-73. doi: 10.1016/j.tiv.2014.06.011. Epub 2014 Jun 30. PMID: 24994473.	Duplicated
105.	Kelber, O., Nieber, K., & Kraft, K. (2014). Valerian: no evidence for clinically relevant interactions. Evidence-Based Complementary and Alternative Medicine, 2014(1), 879396. 10.1155/2014/879396	Review
106.	Zakeri-Milani, P., & Valizadeh, H. (2014). Intestinal transporters: enhanced absorption through P-glycoprotein-related drug interactions. Expert opinion on drug metabolism & toxicology, 10(6), 859-871. 10.1517/17425255.2014.905543	Review
107.	Diallinas, G. (2014). Understanding transporter specificity and the discrete appearance of channel-like gating domains in transporters. Frontiers in pharmacology, 5, 207. 10.3389/fphar.2014.00207	Review
108.	Peters, S., Zimmermann, S., & Adjei, A. A. (2014). Oral epidermal growth factor receptor tyrosine kinase inhibitors for the treatment of non-small cell lung cancer: comparative pharmacokinetics and drug–drug interactions. Cancer treatment Reviews, 40(8), 917-926.	Review
109.	Kruse, V. I. B. E. K. E., Somers, A., Van Bortel, L., De Both, A., Van Belle, S., & Rottey, S. (2014). Sunitinib for metastatic renal cell cancer patients: observational study highlighting the risk of important drug–drug interactions. Journal of clinical pharmacy and therapeutics, 39(3), 259-265. 10.1111/jcpt.12134	Unfocused
110.	Hoy, S. M. (2014). Ponatinib: a Review of its use in adults with chronic myeloid leukaemia or Philadelphia chromosome-positive acute lymphoblastic leukaemia. Drugs, 74(7), 793-806. 10.1007/s40265-014-0216-6	Review
111.	Kast, R. E., Karpel-Massler, G., & Halatsch, M. E. (2014). CUSP9* treatment protocol for recurrent glioblastoma: aprepitant, artesunate, auranofin, captopril, celecoxib, disulfiram, itraconazole, ritonavir, sertraline augmenting continuous low dose temozolomide. Oncotarget, 5(18), 8052. 10.18632/oncotarget.2408	Unfocused
112.	Mandelblatt, J. S., Hurria, A., McDonald, B. C., Saykin, A. J., Stern, R. A., VanMeter, J. W., ... & Ahles, T. (2013, December). Cognitive effects of cancer and its treatments at the intersection of aging: what do we know; what do we need to know?. In Seminars in oncology (Vol. 40, No. 6, pp. 709-725). WB Saunders. 10.1053/j.seminoncol.2013.09.006	Review
113.	Kim, J. H., Choi, A. R., Kim, Y. K., & Yoon, S. (2013). Co-treatment with the anti-malarial drugs mefloquine and primaquine highly sensitizes drug-resistant cancer cells by increasing P-gp inhibition. Biochemical and biophysical research communications, 441(3), 655-660.	Unfocused
114.	Hołysz, H., & Rubiś, B. (2013). Contribution of ABCB1 gene polymorphisms to breast cancer cells response to chemotherapy. Menopause Review/Przegląd Menopauzalny, 12(4), 321-327. 10.5114/pm.2013.37849	Review
115.	Zhang Y, Zhou T, Duan J, Xiao Z, Li G, Xu F. Inhibition of P-glycoprotein and glutathione S-transferase-pi mediated resistance by fluoxetine in MCF-7/ADM cells. Biomed Pharmacother. 2013 Oct;67(8):757-62. doi: 10.1016/j.biopha.2013.04.012. Epub 2013 May 14. PMID: 23731711.	Duplicated
116.	Jat, L. R. (2013). Hyperforin: a potent anti-depressant natural drug. Int. J. Pharm. Pharm. Sci, 5, 9-13.	Review
117.	Grabowsky, J. A. (2013). Drug interactions and the pharmacist: focus on everolimus. Annals of Pharmacotherapy, 47(7-8), 1055-1063. 10.1345/aph.1R769	Review

118.	Thanga Mariappan, T., Mandlekar, S., & Marathe, P. (2013). Insight into tissue unbound concentration: utility in drug discovery and development. Current drug metabolism, 14(3), 324-340. 10.2174/1389200211314030008	Review
119.	Hasan, M. S., Basri, H. B., Hin, L. P., & Stanslas, J. (2013). Genetic polymorphisms and drug interactions leading to clopidogrel resistance: why the Asian population requires special attention. International Journal of Neuroscience, 123(3), 143-154.	Review
120.	Dolghih, E., & Jacobson, M. P. (2013). Predicting efflux ratios and blood-brain barrier penetration from chemical structure: combining passive permeability with active efflux by P-glycoprotein. ACS chemical neuroscience, 4(2), 361-367.10.1021/cn3001922	Unfocused
121.	Parsa, A., Saadati, R., Abbasian, Z., Aramaki, S. A., & Dadashzadeh, S. (2013). Enhanced permeability of etoposide across everted sacs of rat small intestine by vitamin E-TPGS. Iranian journal of pharmaceutical research: IJPR, 12(Suppl), 37.	Unfocused
122.	Billard, C., Merhi, F., & Bauvois, B. (2013). Mechanistic insights into the antileukemic activity of hyperforin. Current cancer drug targets, 13(1), 1-10.	Duplicated
123.	Jia, M. Q., Chen, Z. Y., Wu, X., Liu, J. B., & Wu, Y. F. (2012). Relationshiop between IGF1, IGF1R and Akt and cisplatin resistance of ovarian cancer. Chinese Journal of Cancer Prevention and Treatment, 19(22), 1734-1737.	Unfocused
124.	Peng, B., Gu, Y., Xiong, Y., Zheng, G., & He, Z. (2012). Microarray-assisted pathway analysis identifies MT1X & NFκB as mediators of TCRP1-associated resistance to cisplatin in oral squamous cell carcinoma. PloS one, 7(12), e51413.	Unfocused
125.	Kreuter, J. (2001). Nanoparticulate systems for brain delivery of drugs. Advanced drug delivery Reviews, 47(1), 65-81.	Review
126.	Akamine Y, Yasui-Furukori N, Ieiri I, Uno T. Psychotropic drug-drug interactions involving P-glycoprotein. CNS Drugs. 2012 Nov;26(11):959-73. doi: 10.1007/s40263-012-0008-z. PMID: 23023659.	Duplicated
127.	Le Guellec, C., Benz-de Bretagne, I., Jonville-Bera, A. P., Tarfaoui, N., Andres, C. R., Gendrot, C., & Jourdain, A. (2012). A case of severe toxicity during coadministration of vincristine and piperacillin: are drug transporters involved in vincristine hypersensitivity and drug-drug interactions?. Journal of Pediatric Hematology/Oncology, 34(8), e341-e343. 10.1097/MPH.0b013e318257a4ae	Case
128.	Srivalli, K. M. R., & Lakshmi, P. K. (2012). Overview of P-glycoprotein inhibitors: a rational outlook. Brazilian Journal of Pharmaceutical Sciences, 48, 353-367.	Review
129.	McCaffrey, G., & Davis, T. P. (2012). Physiology and pathophysiology of the blood-brain barrier: P-glycoprotein and occludin trafficking as therapeutic targets to optimize central nervous system drug delivery. Journal of Investigative Medicine, 60(8), 1131-1140.	Review
130.	Ieiri, I. (2012). Functional significance of genetic polymorphisms in P-glycoprotein (MDR1, ABCB1) and breast cancer resistance protein (BCRP, ABCG2). Drug metabolism and pharmacokinetics, 27(1), 85-105. 10.2133/dmpk.DMPK-11-RV-098	Review
131.	Kodaira, H., Kusuhara, H., Fujita, T., Ushiki, J., Fuse, E., & Sugiyama, Y. (2011). Quantitative evaluation of the impact of active efflux by p-glycoprotein and breast cancer resistance protein at the blood-brain barrier on the predictability of the unbound concentrations of drugs in the brain using cerebrospinal fluid concentration as a surrogate. The Journal of Pharmacology and Experimental Therapeutics, 339(3), 935-944. 10.1124/jpet.111.180398	Unfocused
132.	Bikadi, Z., Hazai, I., Malik, D., Jemnid, K., Veres, Z., Hari, P., ... & Mao, Q. (2011). Predicting P-glycoprotein-mediated drug transport based on support vector machine and three-dimensional crystal structure of P-glycoprotein. PloS one, 6(10), e25815. 10.1371/journal.pone.0025815	Unfocused

133.	Sai, K., & Saito, Y. (2011). Ethnic differences in the metabolism, toxicology and efficacy of three anticancer drugs. Expert Opinion on Drug Metabolism & Toxicology, 7(8), 967-988. 10.1517/17425255.2011.585969	Review
134.	Cordier, W., & Steenkamp, V. (2011). Drug interactions in African herbal remedies. 10.1515/DMDI.2011.011	Review
135.	Palmeira A, Rodrigues F, Sousa E, Pinto M, Vasconcelos MH, Fernandes MX. New uses for old drugs: pharmacophore-based screening for the discovery of P-glycoprotein inhibitors. Chem Biol Drug Des. 2011 Jul;78(1):57-72. doi: 10.1111/j.1747-0285.2011.01089.x. Epub 2011 Mar 29. PMID: 21235729.	Duplicated
136.	Deenen, M. J., Cats, A., Beijnen, J. H., & Schellens, J. H. (2011). Part 2: pharmacogenetic variability in drug transport and phase I anticancer drug metabolism. The oncologist, 16(6), 820-834.	Review
137.	Stieger, B., & Meier, P. J. (2011). Pharmacogenetics of drug transporters in the enterohepatic circulation. Pharmacogenomics, 12(5), 611-631. 10.2217/pgs.11.53	Review
138.	Haufroid, V. (2011). Genetic polymorphisms of ATP-binding cassette transporters ABCB1 and ABCC2 and their impact on drug disposition. Current drug targets, 12(5), 631-646. 10.2174/138945011795378487	Review
139.	Wager, T. T., Villalobos, A., Verhoest, P. R., Hou, X., & Shaffer, C. L. (2011). Strategies to optimize the brain availability of central nervous system drug candidates. Expert Opinion on Drug Discovery, 6(4), 371-381. 10.1517/17460441.2011.564158	Review
140.	Ranchon, F., Vantard, N., Gouraud, A., Schwierd, V., Franchon, E., Pham, B. N., ... & Rioufol, C. (2011). Suspicion of drug-drug interaction between high-dose methotrexate and proton pump inhibitors: A Case Report–Should the practice be changed?. Chemotherapy, 57(3), 225-229.	Case
141.	Raizer, J. (2011). Issues in developing drugs for primary brain tumors: barriers and toxicities. Toxicologic pathology, 39(1), 152-157.	Review
142.	Nong, X. L., Li, H., Xia, Y., Li, Y., Li, J. Q., & Yang, Y. P. (2011). In vivo short hairpin RNA interference of vascular endothelial growth factor on drug-resistant tongue cancer cells. Zhonghua kou Qiang yi xue za zhi= Zhonghua Kouqiang Yixue Zazhi= Chinese Journal of Stomatology, 46(1), 15-19.	Unfocused
143.	Grimberg, B. T., & Mehlotra, R. K. (2011). Expanding the antimalarial drug arsenal—Now, but how?. Pharmaceuticals, 4(5), 681-712. 10.3390/ph4050681	Review
144.	Kleine-Brueggeney, M., Musshoff, F., Stuber, F., & Stamer, U. M. (2010). Pharmacogenetics in palliative care. Forensic science international, 203(1-3), 63-70. 10.1016/j.forsciint.2010.07.003	Review
145.	Tarirai, C., Viljoen, A. M., & Hamman, J. H. (2010). Herb–drug pharmacokinetic interactions Reviewed. Expert opinion on drug metabolism & toxicology, 6(12), 1515-1538. 10.1517/17425255.2010.529129	Review
146.	Parker, K. A., Glaysher, S., Polak, M., Gabriel, F. G., Johnson, P., Knight, L. A., ... & Cree, I. A. (2010). The molecular basis of the chemosensitivity of metastatic cutaneous melanoma to chemotherapy. Journal of clinical pathology, 63(11), 1012-1020. 10.1136/jcp.2010.080119	Unfocused
147.	Longo, R., D'Andrea, M., Sarmiento, R., & Gasparini, G. (2010). Pharmacogenetics in breast cancer: focus on hormone therapy, taxanes, trastuzumab and bevacizumab. Expert Opinion on Investigational Drugs, 19(sup1), S41-S50. 10.1517/13543781003732701	Review
148.	Yee, S. W., Chen, L., & Giacomini, K. M. (2010). Pharmacogenomics of membrane transporters: past, present and future. Pharmacogenomics, 11(4), 475-479. 10.2217/pgs.10.22	Editorial
149.	Ishikawa, T., Sakurai, A., Hirano, H., Lezhava, A., Sakurai, M., & Hayashizaki, Y. (2010). Emerging New Technologies in Pharamcogenomics: Rapid SNP detection, molecular dynamic simulation, and QSAR analysis methods to validate clinically important genetic	Review

	variants of human ABC Transporter ABCB1 (P-gp/MDR1). Pharmacology & therapeutics, 126(1), 69-81. 10.1016/j.pharmthera.2010.01.005	
150.	Kiyotani, K., Mushiroda, T., Imamura, C. K., Hosono, N., Tsunoda, T., Kubo, M., ... & Zembutsu, H. (2010). Significant effect of polymorphisms in CYP2D6 and ABCC2 on clinical outcomes of adjuvant tamoxifen therapy for breast cancer patients. Journal of Clinical Oncology, 28(8), 1287-1293.	Unfocused
151.	Broccatelli, F., Carosati, E., Cruciani, G., & Oprea, T. I. (2010). Transporter-mediated efflux influences CNS side effects: ABCB1, from antitarget to target. Molecular Informatics, 29(1-2), 16-26. 10.1002/minf.200900075	Review
152.	Palmer, A. M. (2010). The role of the blood–CNS barrier in CNS disorders and their treatment. Neurobiology of disease, 37(1), 3-12. 10.1016/j.nbd.2009.07.029	Review
153.	Nicolazzo, J. A., & Mehta, D. C. (2010). Transport of Drugs Across the blood–brain Barrier in Alzheimer's Disease. Therapeutic delivery, 1(4), 595-611. 10.4155/tde.10.41	Review
154.	Ferner, R. E. (2010). ABCB1 (P-glycoprotein) and the clinical pharmacology of adverse drug reactions. Adverse Drug Reaction Bulletin, (263), 1011-1014. 10.1097/FAD.0b013e32833ee68a	Review
155.	Sarginson, J. E., Lazzeroni, L. C., Ryan, H. S., Ershoff, B. D., Schadberg, A. F., & Murphy Jr, G. M. (2010). ABCB1 (MDR1) polymorphisms and antidepressant response in geriatric depression. Pharmacogenetics and genomics, 20(8), 467-475. 10.1097/FPC.0b013e32833b593a	Duplicated
156.	Stanley, L. A., Horsburgh, B. C., Ross, J., Scheer, N., & Wolf, C. R. (2009). Drug transporters: gatekeepers controlling access of xenobiotics to the cellular interior. Drug metabolism Reviews, 41(1), 27-65. 10.1080/03602530802605040	Review
157.	Krčevski-Škvarč, N. (2009). Variability in the response to opioid therapy. Libri Oncologici: Croatian Journal of Oncology, 37(1-3), 43-46.	Review
158.	Ueno, M. (2009). Mechanisms of the penetration of blood-borne substances into the brain. Current Neuropharmacology, 7(2), 142-149. 10.2174/157015909788848901	Review
159.	Vähäkangas, K., & Myllynen, P. (2009). Drug transporters in the human blood-placental barrier. British journal of pharmacology, 158(3), 665-678. 10.1111/j.1476-5381.2009.00336.x	Review
160.	Ravna, A. W., Sager, G., Dahl, S. G., & Sylte, I. (2008). Membrane transporters: structure, function and targets for drug design. In Transporters as targets for drugs (pp. 15-51). Berlin, Heidelberg: Springer Berlin Heidelberg. 10.1007/7355_2008_023	Review
161.	Alibert-Franco, S., Pradines, B., Mahamoud, A., & Davin-Regli, A. (2009). Efflux mechanism, an attractive target to combat multidrug resistant Plasmodium falciparum and Pseudomonas aeruginosa. Current medicinal chemistry, 16(3), 301-317. 10.2174/092986709787002619	Review
162.	Xiang, C. D., Batugo, M., Gale, D. C., Zhang, T., Ye, J., Li, C., ... & Zhang, E. Y. (2009). Characterization of human corneal epithelial cell model as a surrogate for corneal permeability assessment: metabolism and transport. Drug Metabolism and Disposition, 37(5), 992-998. 10.1124/dmd.108.026286	Unfocused
163.	Nakagawa, S., Deli, M. A., Kawaguchi, H., Shimizudani, T., Shimono, T., Kittel, A., ... & Niwa, M. (2009). A new blood–brain barrier model using primary rat brain endothelial cells, pericytes and astrocytes. Neurochemistry international, 54(3-4), 253-263. 10.1016/j.neuint.2008.12.002	Unfocused
164.	Mouly, S., Meune, C., & Bergmann, J. F. (2009). Mini-series: I. Basic science. Uncertainty and inaccuracy of predicting CYP-mediated in vivo drug interactions in the ICU from in vitro models: focus on CYP3A4. Intensive care medicine, 35(3), 417-429. 10.1007/s00134-008-1384-1	Review
165.	Argov M, Kashi R, Peer D, Margalit R. Treatment of resistant human colon cancer xenografts by a fluoxetine-doxorubicin combination enhances therapeutic responses	Duplicated

	comparable to an aggressive bevacizumab regimen. Cancer Lett. 2009 Feb 8;274(1):118-25. doi: 10.1016/j.canlet.2008.09.005. Epub 2008 Oct 11. PMID: 18851896.	
166.	Guessous, I., Gwinn, M., Yu, W., Yeh, J., Clyne, M., & Khoury, M. J. (2009). Trends in pharmacogenomic epidemiology: 2001–2007. Public Health Genomics, 12(3), 142-148. 10.1159/000189626	Review
167.	Mini, E., & Nobili, S. (2009). Pharmacogenetics: implementing personalized medicine. Clinical cases in mineral and bone metabolism, 6(1), 17.	Review
168.	von Richter, O., Glavinas, H., Krajcsi, P., Liehner, S., Siewert, B., & Zech, K. (2009). A novel screening strategy to identify ABCB1 substrates and inhibitors. Naunyn-Schmiedeberg's archives of pharmacology, 379(1), 11-26.	Unfocused
169.	Huls, M., Russel, F. G., & Masereeuw, R. (2009). The role of ATP binding cassette transporters in tissue defense and organ regeneration. The Journal of pharmacology and experimental therapeutics, 328(1), 3-9. 10.1124/jpet.107.132225	Review
170.	Mealey, K. L. (2008). Canine ABCB1 and macrocyclic lactones: heartworm prevention and pharmacogenetics. Veterinary Parasitology, 158(3), 215-222. 10.1016/j.vetpar.2008.09.009	Review
171.	Dauchy, S., Tournier, N., Yousif, S., Jacob, A., & Declèves, X. (2008). Barrière hémato-encéphalique: implication des transporteurs ABC en neuropharmacologie. Réanimation, 17(7), 664-669. 10.1016/j.reaurg.2008.07.013	Review
172.	Palumbo, P., Eramo, A., & DE BARTOLOMEIS, A. (2008). Janus's face of schizophrenia treatment: neurobiology of antipsychotic treatments response and resistance. Rivista di Psichiatria, 43(5), 269-291.	Review
173.	Szakács, G., Váradi, A., Özvegy-Laczka, C., & Sarkadi, B. (2008). The role of ABC transporters in drug absorption, distribution, metabolism, excretion and toxicity (ADME–Tox). Drug discovery today, 13(9-10), 379-393. 10.1016/j.drudis.2007.12.010	Review
174.	Funk, C. (2008). The role of hepatic transporters in drug elimination. Expert opinion on drug metabolism & toxicology, 4(4), 363-379. 10.1517/17425255.4.4.363	Review
175.	Wang, J. S., Newport, D. J., Stowe, Z. N., Donovan, J. L., Pennell, P. B., & DeVane, C. L. (2007). The emerging importance of transporter proteins in the psychopharmacological treatment of the pregnant patient. Drug metabolism Reviews, 39(4), 723-746. 10.1080/03602530701690390	Review
176.	Aszalos, A. (2007). Drug–drug interactions affected by the transporter protein, P-glycoprotein (ABCB1, MDR1): II. Clinical aspects. Drug discovery today, 12(19-20), 838-843. 10.1016/j.drudis.2007.07.022	Review
177.	Calcagno, A. M., Kim, I. W., Wu, C. P., Shukla, S., & Ambudkar, S. V. (2007). ABC drug transporters as molecular targets for the prevention of multidrug resistance and drug-drug interactions. Current drug delivery, 4(4), 324-333. 10.2174/156720107782151241	Review
178.	Altinoz, M. A., Gedikoglu, G., Sav, A., Ozcan, E., Ozdilli, K., Bilir, A., & Del Maestro, R. F. (2007). Medroxyprogesterone acetate induces c6 glioma chemosensitization via antidepressant-like lysosomal phospholipidosis/myelinosis in vitro. International Journal of Neuroscience, 117(10), 1465-1480. 10.1080/00207450701540062	Duplicated
179.	Watson, M. B., Lind, M. J., & Cawkwell, L. (2007). Establishment of in-vitro models of chemotherapy resistance. Anti-Cancer Drugs, 18(7), 749-754. 10.1097/CAD.0b013e3280a02f43	Review
180.	Ponte-Sucre, A. (2007). Availability and applications of A TP-binding c assette (ABC) transporter blockers. Applied microbiology and biotechnology, 76(2), 279-286. 10.1007/s00253-007-1017-6	Review
181.	Kristiansen, J. E., Hendricks, O., Delvin, T., Butterworth, T. S., Aagaard, L., Christensen, J. B., ... & Keyzer, H. (2007). Reversal of resistance in microorganisms by help of non-antibiotics. Journal of Antimicrobial Chemotherapy, 59(6), 1271-1279. 10.1093/jac/dkm071	Review

182.	van Breemen, M. S., Wilms, E. B., & Vecht, C. J. (2007). Epilepsy in patients with brain tumours: epidemiology, mechanisms, and management. The Lancet Neurology, 6(5), 421-430.	Review
183.	Ueno, M. (2007). Molecular anatomy of the brain endothelial barrier: an overview of the distributional features. Current medicinal chemistry, 14(11), 1199-1206.	Review
184.	Petrovic, V., Teng, S., & Piquette-Miller, M. (2007). Regulation of drug transporters: during infection and inflammation. Molecular interventions, 7(2), 99.	Review
185.	Gebhart, B. C., Barker, B. C., & Markewid, B. A. (2007). Decreased serum linezolid levels in a critically ill patient receiving concomitant linezolid and rifampin. Pharmacotherapy: The Journal of Human Pharmacology and Drug Therapy, 27(3), 476-479. 10.1592/phco.27.3.476	Case
186.	Lu, R., Sun, J., Zhao, C., & He, Z. (2007). Transporters: significant roles in drug transport at blood-cerebrospinal fluid barrier. JOURNAL-CHINA PHARMACEUTICAL UNIVERSITY, 38(1), 92.	Review
187.	Besag, F. M. (2007). Is current drug safety an issue?. Current Drug Safety, 2(1), 1-4.	Editorial
188.	Gao, G. L., Wan, H. Y., Zou, X. S., Chen, W. X., Chen, Y. Q., & Huang, X. Z. (2007). Relationship between the expression of P-glycoprotein, glutathione S-transferase-pi and thymidylate synthase proteins and adenosine triphosphate tumor chemosensitivity assay in cervical cancer. Zhonghua fu chan ke za zhi, 42(3), 201-205.	Duplicated
189.	Dowling, P. (2006). Pharmacogenetics: it's not just about ivermectin in collies. The Canadian Veterinary Journal, 47(12), 1165.	Review
190.	Hardiman, G. (2006). Microarrays Technologies 2006: an overview. Pharmacogenomics, 7(8), 1153-1158.	Review
191.	Engen, R. M., Marsh, S., Van Booven, D. J., & McLeod, H. L. (2006). Ethnic differences in pharmacogenetically relevant genes. Current drug targets, 7(12), 1641-1648.	Review
192.	Pal, D., & Mitra, A. K. (2006). CYP3A4 and MDR mediated interactions in drug therapy. Clinical Research and Regulatory Affairs, 23(3-4), 125-163. 10.1080/10601330600961935	Review
193.	Zhang, P., Zhang, Z., Zhou, X., Qiu, W., Chen, F., & Chen, W. (2006). Identification of genes associated with cisplatin resistance in human oral squamous cell carcinoma cell line. BMC cancer, 6(1), 224. 10.1186/1471-2407-6-224	Unfocused
194.	Alisky, J. M., Chertkova, E. L., & Iczkowski, K. A. (2006). Drug interactions and pharmacogenetic reactions are the basis for chloroquine and mefloquine-induced psychosis. Medical hypotheses, 67(5), 1090-1094.	Editorial
195.	Michalak, K., Wesolowska, O., Motohashi, N., Molnar, J., & Hendrich, A. B. (2006). Interactions of phenothiazines with lipid bilayer and their role in multidrug resistance reversal. Current drug targets, 7(9), 1095-1105. 10.2174/138945006778226570	Review
196.	Tsakovska I. Phenothiazines and structurally related compounds as modulators of cancer multidrug resistance. Current Drug Targets. 09.2006;7(9):1123-1134. doi: 10.2174/138945006778226660.	Review
197.	Balayssac D. Involvement of the multidrug resistance transporters in cisplatin-induced neuropathy in rats. comparison with the chronic constriction injury model and monoarthritic rats. European journal of pharmacology. 08.2006;544(1-3):49-57. doi: 10.1016/j.ejphar.2006.06.055.	Duplicated
198.	Choudhuri S. Structure, function, expression, genomic organization, and single nucleotide polymorphisms of human ABCB1 (MDR1), ABCC (MRP), and ABCG2 (BCRP) efflux transporters. International journal of toxicology. 07.2006;25(4):231-259. doi: 10.1080/10915810600746023.	Review
199.	Annese V. Multidrug resistance 1 gene in inflammatory bowel disease: A meta-analysis. World Journal of Gastroenterology. 2006;12(23):3636-3644. doi: 10.3748/wjg.v12.i23.3636.	Review
200.	Peer D. Fluoxetine and reversal of multidrug resistance. Cancer letters. 06.2006;237(2):180-187. doi: 10.1016/j.canlet.2005.06.003.	Review

201.	Pal D. MDR- and CYP3A4-mediated drug–herbal interactions. Life sciences (1973). 03.2006;78(18):2131-2145. doi: 10.1016/j.lfs.2005.12.010.	Duplicated
202.	Gatlik-Landwojtowicz E. Quantification and characterization of P-Glycoprotein−Substrate interactions. Biochemistry (Easton). 03.2006;45(9):3020-3032. doi: 10.1021/bi051380+.	Unfocused
203.	Schulz V. Safety of st. john's wort extract compared to synthetic antidepressants. Phytomedicine (Stuttgart). 02.2006;13(3):199-204. doi: 10.1016/j.phymed.2005.07.005.	Duplicated
204.	Quiney C. Hyperforin, a new lead compound against the progression of cancer and leukemia? Leukemia. 09.2006;20(9):1519-1525. doi: 10.1038/sj.leu.2404301.	Review
205.	Crivori P. Computational models for identifying potential P-glycoprotein substrates and inhibitors. Molecular Pharmaceutics. 02.2006;3(1):33-44. doi: 10.1021/mp050071a.	Unfocused
206.	Tandon V. P-glycoprotein: Pharmacological relevance. Indian journal of pharmacology. 2006;38(1):13-24. doi: 10.4103/0253-7613.19847.	Review
207.	Sun H. Effects of renal failure on drug transport and metabolism. Pharmacology & therapeutics (Oxford). 01.2006;109(1-2):1-11. doi: 10.1016/j.pharmthera.2005.05.010.	Review
208.	Panchagnula R. Co-treatment with grapefruit juice inhibits while chronic administration activates intestinal P-glycoprotein-mediated drug efflux. Pharmazie. 2005;60(12):922-927.	Duplicated
209.	Pajeva I. Molecular modeling of P-glycoprotein and related drugs. Medicinal chemistry research. 02.2005;14(2):106-117. doi: 10.1007/s00044-005-0127-x.	Unfocused
210.	Becquemont L. Pharmacogenetics in daily medical practice | la pharmacogénétique dans l'exercice médical quotidien. Medecine Therapeutique. 2005;11(6):437-445.	Review
211.	Sakurai A. Genetic polymorphisms of ATP-binding cassette transporters ABCB1 and ABCG2: Therapeutic implications. Expert Opinion on Pharmacotherapy. 11.2005;6(14):2455-2473. doi: 10.1517/14656566.6.14.2455.	Review
212.	Löscher W. Drug resistance in brain diseases and the role of drug efflux transporters. Nature Reviews. Neuroscience. 08.2005;6(8):591-602. doi: 10.1038/nrn1728.	Review
213.	Sheffield LJ. The second annual meeting of the international society of pharmacogenomics (ISP)—a joint meeting with the pacific rim association for clinical pharmacogenetics (PRACPG). The pharmacogenomics journal. 06.2005;5(3):144-145. doi: 10.1038/sj.tpj.6500310.	Review
214.	Sheffield LJ. The third annual meeting of the international society of pharmacogenomics (ISP): A joint meeting with the international union of nutritional sciences (IUNS) task force on genetics and nutrition. The pharmacogenomics journal. 06.2005;5(3):146-148. doi: 10.1038/sj.tpj.6500311.	Review
215.	Abnormal drug response: Opportunities for risk reduction through pharmacogenetics. WHO drug information. 2005;19(1):3-12.	Review
216.	Beringer PM. Transporters and their impact on drug disposition. The Annals of pharmacotherapy. 06.2005;39(6):1097-1108. doi: 10.1345/aph.1e614.	Review
217.	Löscher W. Role of drug efflux transporters in the brain for drug disposition and treatment of brain diseases. Progress in neurobiology. 05.2005;76(1):22-76. doi: 10.1016/j.pneurobio.2005.04.006.	Review
218.	Lepper ER. Mechanisms of resistance to anticancer drugs: The role of the polymorphic ABC transporters ABCB1 and ABCG2. Pharmacogenomics. 03.2005;6(2):115-138. doi: 10.1517/14622416.6.2.115.	Review
219.	Wang Q. Evaluation of the MDR-MDCK cell line as a permeability screen for the blood–brain barrier. International journal of pharmaceutics. 01.2005;288(2):349-359. doi: 10.1016/j.ijpharm.2004.10.007.	Unfocused
220.	Löscher W. Blood-brain barrier active efflux transporters: ATP-binding cassette gene family. NeuroRx. 01.2005;2(1):86-98. doi: 10.1602/neurorx.2.1.86.	Review

221.	Elsinga PH. Positron emission tomography studies on binding of central nervous system drugs and P-glycoprotein function in the rodent brain. Molecular imaging and biology. 01.2005;7(1):37-44. doi: 10.1007/s11307-005-0951-x.	Review
222.	Bachmeier CJ. A fluorometric screening assay for drug efflux transporter activity in the blood-brain barrier. Pharmaceutical research. 01.2005;22(1):113-121. doi: 10.1007/s11095-004-9016-0.	Unfocused
223.	De Giorgi U. Imatinib and gastrointestinal stromal tumors: Where do we go from here? Molecular Cancer Therapeutics. 03.2005;4(3):495-501. doi: 10.1158/1535-7163.mct-04-0302.	Unfocused
224.	Hoffmann U. The ABC transporters MDR1 and MRP2: Multiple functions in disposition of xenobiotics and drug resistance. Drug Metabolism Reviews. 01.2004;36(3-4):669-701. doi: 10.1081/dmr-200033473.	Review
225.	Peer D. Fluoxetine inhibits multidrug resistance extrusion pumps and enhances responses to chemotherapy in syngeneic and in human xenograft mouse tumor models. Cancer research (Chicago, Ill.). 10.2004;64(20):7562-7569. doi: 10.1158/0008-5472.can-03-4046.	Duplicated
226.	DELANGE E. Potential role of ABC transporters as a detoxification system at the blood?CSF barrier. Advanced drug delivery Reviews. 10.2004;56(12):1793-1809. doi: 10.1016/j.addr.2004.07.009.	Review
227.	Nicolantonio FD. Ex vivo characterization of XR11576 (MLN576) against ovarian cancer and other solid tumors. Anti-cancer drugs. 10.2004;15(9):849-860. doi: 10.1097/00001813-200410000-00005.	Unfocused
228.	Mealey KL. Therapeutic implications of the MDR-1 gene. Journal of veterinary pharmacology and therapeutics. 10.2004;27(5):257-264. doi: 10.1111/j.1365-2885.2004.00607.x.	Review
229.	Wiersma. The importance of the right drug for the right patient: Pharmacogenetics in the pediatric practice. Tijdschrift voor Kindergeneeskunde. 2004;72(5):190-197.	Review
230.	Zhou S. Therapeutic drugs that behave as mechanism-based inhibitors of cytochrome P450 3A4. Current Drug Metabolism. 10.2004;5(5):415-442. doi: 10.2174/1389200043335450.	Duplicated
231.	Mannel M. Drug interactions with st john???s wort mechanisms and clinical implications. Drug safety. 2004;27(11):773-797. doi: 10.2165/00002018-200427110-00003.	Duplicated
232.	Ieiri I. The MDR1 (ABCB1) gene polymorphism and its clinical implications. Clinical pharmacokinetics. 2004;43(9):553-576. doi: 10.2165/00003088-200443090-00001.	Review
233.	Fromm MF. Importance of P-glycoprotein at blood–tissue barriers. Trends in pharmacological sciences (Regular ed.). 08.2004;25(8):423-429. doi: 10.1016/j.tips.2004.06.002.	Review
234.	Lavedan C. Translating the genome into individualized therapeutics. Drug development research. 08.2004;62(4):371-382. doi: 10.1002/ddr.10390.	Review
235.	Syme MR. Drug transfer and metabolism by the human placenta. Clinical pharmacokinetics. 2004;43(8):487-514. doi: 10.2165/00003088-200443080-00001.	Review
236.	Frueh FW. From pharmacogenetics to personalized medicine: A vital need for educating health professionals and the community. Pharmacogenomics. 07.2004;5(5):571-579. doi: 10.1517/14622416.5.5.571.	Review
237.	Elsinga P. PET studies on P-glycoprotein function in the blood-brain barrier: How it affects uptake and binding of drugs within the CNS. Current pharmaceutical design. 05.2004;10(13):1493-1503. doi: 10.2174/1381612043384736.	Review
238.	Ingels F. Effect of simulated intestinal fluid on drug permeability estimation across caco-2 monolayers. International journal of pharmaceutics. 04.2004;274(1-2):221-232. doi: 10.1016/j.ijpharm.2004.01.014.	Unfocused
239.	Nagy H. Distinct groups of multidrug resistance modulating agents are distinguished by competition of P-glycoprotein-specific antibodies. Biochemical and biophysical research communications. 03.2004;315(4):942-949. doi: 10.1016/j.bbrc.2004.01.156.	Unfocused

240.	Lee C. Pharmacogenetics of the human MDR1 multidrug transporter. Current Pharmacogenomics. 03.2004;2(1):1-11. doi: 10.2174/1570160043476097.	Review
241.	Marzolini C. Polymorphisms in human MDR1 (P-glycoprotein): Recent advances and clinical relevance. Clinical pharmacology and therapeutics. 01.2004;75(1):13-33. doi: 10.1016/j.clpt.2003.09.012.	Review
242.	Nicolantonio FD. Ex vivo reversal of chemoresistance by tariquidar (XR9576). Anti-cancer drugs. 10.2004;15(9):861-869. doi: 10.1097/00001813-200410000-00006.	Duplicated
243.	Pauli-Magnus C. Functional implications of genetic polymorphisms in the multidrug resistance gene MDR1 (ABCB1). Pharmaceutical research. 06.2004;21(6):904-913. doi: 10.1023/b:pham.0000029276.21063.0b.	Review
244.	Zhou C. Correlation between p53 gene mutation and the expression of tumor drug resistance genes in lung cancer and its clinical significance. Zhonghua jie he he hu xi za zhi = Zhonghua jiehe he huxi zazhi = Chinese journal of tuberculosis and respiratory diseases. 2004;27(10):678-682.	Duplicated
245.	Schwab M. Genetic polymorphisms of the HumanMDR1Drug transporter. Annual Review of pharmacology and toxicology. 04.2003;43(1):285-307. doi: 10.1146/annurev.pharmtox.43.100901.140233.	Review
246.	Barbieri F. Quinolizidinyl derivatives of iminodibenzyl and phenothiazine as multidrug resistance modulators in ovarian cancer cells. Investigational new drugs. 11.2003;21(4):413-420. doi: 10.1023/a:1026295017158.	Duplicated
247.	Ambudkar SV. P-glycoprotein: From genomics to mechanism. Oncogene. 10.2003;22(47):7468-7485. doi: 10.1038/sj.onc.1206948.	Review
248.	Varma M. P-glycoprotein inhibitors and their screening: A perspective from bioavailability enhancement. Pharmacological research. 10.2003;48(4):347-359. doi: 10.1016/s1043-6618(03)00158-0.	Review
249.	Kolaczkowski M. Phenothiazines as potent modulators of yeast multidrug resistance. International journal of antimicrobial agents. 09.2003;22(3):279-283. doi: 10.1016/s0924-8579(03)00214-0.	Unfocused
250.	Nagasubramanian R. Pharmacogenomics – racing towards personalized prescriptions. Laboratory medicine. 09.2003;34(9):651-659. doi: 10.1309/fl6qkvfvh6p7nv5e.	Review
251.	Li Q. Influence of drugs and nutrients on transporter gene expression levels in caco-2 and LS180 intestinal epithelial cell lines. Pharmaceutical research. 08.2003;20(8):1119-1124. doi: 10.1023/a:1025076326061.	Unfocused
252.	Martel, F., & Azevedo, I. (2003). An update on the extraneuronal monoamine transporter (EMT): characteristics, distribution and regulation. Current Drug Metabolism, 4(4), 313-318. 10.2174/1389200033489433	Review
253.	Langer Pathways to individual drug therapy 2003	Review
254.	Hendrich, A. B., & Michalak, K. (2003). Lipids as a target for drugs modulating multidrug resistance of cancer cells. Current drug targets, 4(1), 23-30. 10.2174/1389450033347172	Review
255.	Geubel, A. P. (2002). La toxicité hépatique des médicaments. Louvain médical, 121(9), S186-S191.	Review
256.	Carson, S. W., Ousmanou, A. D., & Hoyler, S. L. (2002). Emerging significance of P-glycoprotein in understanding drug disposition and drug interactions in psychopharmacology. Psychopharmacology Bulletin, 36(1), 67-81.	Duplicated
257.	Laska, D. A., Houchins, J. O., Pratt, S. E., Horn, J., Xia, X., Hanssen, B. R., ... & Lindstrom, T. (2002). Characterization and application of a vinblastine-selected Caco-2 cell line for evaluation of P-glycoprotein. In Vitro Cellular & Developmental Biology-Animal, 38(7), 401-410. 10.1290/1071-2690(2002)038<0401:CAAOAV>2.0.CO;2	Unfocused
258.	Boven, E. (2002). Pharmacokinetics in Cancer Treatment: Clinical Implications of Interindividual Variability and Drug Interactions. American Journal of Cancer, 1(1), 33-53.	Review

259.	Davis, A., & Long, R. (2001). Pharmacogenetics research network and knowledge base: 1st annual scientific meeting. Pharmacogenomics, 2(3), 285-289. 10.1517/14622416.2.3.285	Review
260.	Arvelo, F., & Merentes, E. (2001). Pharmacological biomodulation in cancer. Acta Cientifica Venezolana, 52(1), 68-77.	Review
261.	Szabó, D., Keyzer, H., Kaiser, H. E., & Molnár, J. (2000). Reversal of multidrug resistance of tumor cells. Anticancer research, 20(6B), 4261-4274.	Review
262.	Drewe, J., & Krähenbühl, S. (2000). Clinical pharmacology: proteins for the transport of drugs. Schweizerische Medizinische Wochenschrift, 130(20), 727-731.	Review
263.	Liu, Y., & Hu, M. (2000). P-glycoprotein and bioavailability-implication of polymorphism. 10.1515/CCLM.2000.127	Duplicated
264.	Szabó, D., Szabó Jr, G., Ocsovszki, I., Aszalos, A., & Molnár, J. (1999). Anti-psychotic drugs reverse multidrug resistance of tumor cell lines and human AML cells ex-vivo. Cancer letters, 139(1), 115-119. 10.1016/S0304-3835(99)00020-8	Included
265.	Ibrahim, S., Knapton, A., Licht, T., & Aszalos, A. (1999). Influence of antipsychotic, antiemetic, and antifungal drugs of the function of P-glycoprotein in Caco-2 cells. Clinical Pharmacology & Therapeutics, 65(2), 199-199. 10.1016/S0009-9236(99)80324-5	Unfocused
266.	Bray, P. G., & Ward, S. A. (1998). A comparison of the phenomenology and genetics of multidrug resistance in cancer cells and quinoline resistance in Plasmodium falciparum. Pharmacology & therapeutics, 77(1), 1-28. 10.1016/S0163-7258(97)00083-1	Review
267.	Hunter, J., & Hirst, B. H. (1997). Intestinal secretion of drugs. The role of P-glycoprotein and related drug efflux systems in limiting oral drug absorption. Advanced drug delivery Reviews, 25(2-3), 129-157. 10.1016/S0169-409X(97)00497-3	Review
268.	Hegewisch-Becker, S. (1996). MDR1 reversal: criteria for clinical trials designed to overcome the multidrug resistance phenotype. Leukemia, 10, S32-S38. PMID: 8615610	Review
269.	Varga, A., Nugel, H., Baehr, R., Marx, U., Hevér, A., Nacsa, J., ... & Molnár, J. (1996). Reversal of multidrug resistance by amitriptyline in vitro. Anticancer research, 16(1), 209-211.	Unfocused
270.	Pajeva, I. K., Wiese, M., Cordes, H. P., & Seydel, J. K. (1996). Membrane interactions of some catamphiphilic drugs and relation to their multidrug-resistance-reversing ability. Journal of cancer research and clinical oncology, 122(1), 27-40. 10.1007/BF01203070	Duplicated
271.	Dianzani, F., Turriziani, O., Riva, E., Bambacioni, F., Solmone, M., Simeoni, E., ... & Antonelli, G. (1995). Cellular factors leading to AZT resistance in HIV.	Unfocused
272.	Jaffrézou, J. P., Chen, G., Durán, G. E., Muller, C., Bordier, C., Laurent, G., ... & Levade, T. (1995). Inhibition of lysosomal acid sphingomyelinase by agents which reverse multidrug resistance. Biochimica et Biophysica Acta (BBA)-Molecular Cell Research, 1266(1), 1-8. 10.1016/0167-4889(94)00219-5	Duplicated
273.	Chapman, A. E., & Goldstein, L. J. (1995, February). Multiple drug resistance: biologic basis and clinical significance in renal-cell carcinoma. In Seminars in oncology (Vol. 22, No. 1, pp. 17-28).	Review
274.	Lyubimov, E., Lan, L. B., Pashinsky, I., Ayesh, S., & Stein, W. D. (1995). Saturation reversal of the multidrug pump using many reversers in low-dose combinations. Anti-cancer drugs, 6(6), 727-735. 10.1097/00001813-199512000-00003	Unfocused
275.	Licht, T., Pastan, I., Gottesman, M., & Herrmann, F. (1994). P-glycoprotein-mediated multidrug resistance in normal and neoplastic hematopoietic cells. Annals of hematology, 69(4), 159-171. 10.1007/BF02215949	Review
276.	Ferry, D. R., & Kerr, D. J. (1994). Multidrug resistance in cancer. Bmj, 308(6922), 148-149. 10.1136/bmj.308.6922.148	Editorial
277.	Lehnert, M. (1994). Reversal of P-glycoprotein-associated multidrug resistance: from bench to bedside. Oncology Research and Treatment, 17(1), 8-15. 10.1159/000218375	Review

278.	Wadkins, R. M., & Houghton, P. J. (1993). The role of drug-lipid interactions in the biological activity of modulators of multi-drug resistance. Biochimica et Biophysica Acta (BBA)-Biomembranes, 1153(2), 225-236. 10.1016/0005-2736(93)90409-S	Unfocused
279.	Kellen, J. A. (1993). Reversal of multidrug resistance in cancer.	Review
280.	Lum, B. L., Fisher, G. A., Brophy, N. A., Yahanda, A. M., Adler, K. M., Kaubisch, S., ... & Sikic, B. I. (1993). Clinical trials of modulation of multidrug resistance pharmacokinetic and pharmacodynamic considerations. Cancer, 72(S11), 3502-3514. 10.1002/1097-0142(19931201)72:11+<3502::AID-CNCR2820721618>3.0.CO;2-N	Unfocused
281.	Walter, R. D., Seth, M., & Bhaduri, A. P. (1993). Reversal of chloroquine resistance in Plasmodium falciparum by CDR 87/209 and analogues. Tropical Medicine and Parasitology: Official Organ of Deutsche Tropenmedizinische Gesellschaft and of Deutsche Gesellschaft fur Technische Zusammenarbeit (GTZ), 44(1), 5-8.	Unfocused
282.	Molnár, J., Tarodi, B., Galfi, M., Matkovics, B., & Motohashi, N. (1992). In vitro antiproliferative effects of tricyclic psychopharmaceutical agents and synergism with some resistance modifiers. Anticancer research, 12(1), 273-280.	Unfocused
283.	Miyamoto, K., Wakusawa, S., Nakamura, S., Tajima, K., & Hidaka, H. (1992). Multidrug resistance in Yoshida rat ascites hepatoma cell lines. Anticancer research, 12(3), 649-653.	Unfocused
284.	Fardel, O., Ratanasavanh, D., Loyer, P., Ketterer, B., & Guillouzo, A. (1992). Overexpression of the multidrug resistance gene product in adult rat hepatocytes during primary culture. European journal of biochemistry, 205(2), 847-852. 10.1111/j.1432-1033.1992.tb16849.x	Unfocused
285.	Watt, G., Long, G. W., Grogl, M., & Martin, S. K. (1990). Reversal of drug-resistant falciparum malaria by calcium antagonists: potential for host cell toxicity. Transactions of the Royal Society of Tropical Medicine and Hygiene, 84(2), 187-190. 10.1016/0035-9203(90)90248-D	Unfocused
286.	Ford, J. M., Prozialeck, W. C., & Hait, W. N. (1989). Structural features determining activity of phenothiazines and related drugs for inhibition of cell growth and reversal of multidrug resistance. Molecular pharmacology, 35(1), 105-115.	Unfocused

Table S3. Results from the PsycInfo database search with final decision label.		
1.	Aguilar E, Monreal JA, Palao DJ. Pharmacogenetics in psychiatry: Clinical case of resistant depression and a previous history of multiple adverse effects. Actas Esp Psiquiatr. 2017;45(6):303-306.	Case
2.	Chaves C, Remiao F, Cisternino S, Decleves X. Opioids and the Blood-Brain Barrier: A Dynamic Interaction with Consequences on Drug Disposition in Brain. Curr Neuropharmacol. 2017;15(8):1156-1173. doi:10.2174/1570159X15666170504095823	Review
3.	Chang HH, Chou CH, Yang YK, Lee IH, Chen PS. Association between ABCB1 Polymorphisms and Antidepressant Treatment Response in Taiwanese Major Depressive Patients. Clin Psychopharmacol Neurosci. 2015;13(3):250-255. doi:10.9758/cpn.2015.13.3.250	Unfocused
4.	Akamine Y, Yasui-Furukori N, Ieiri I, Uno T. Psychotropic drug-drug interactions involving P-glycoprotein. CNS Drugs. 2012;26(11):959-973. doi:10.1007/s40263-012-0008-z	Duplicated
5.	Mason BL, Thomas SA, Lightman SL, Pariante CM. Desipramine treatment has minimal effects on the brain accumulation of glucocorticoids in P-gp-deficient and wild-type mice. Psychoneuroendocrinology. 2011;36(9):1351-1360. doi:10.1016/j.psyneuen.2011.03.008	Unfocused
6.	Ruike Z, Junhua C, Wenxing P. In vitro and in vivo evaluation of the effects of duloxetine on P-gp function. Hum Psychopharmacol. 2010;25(7-8):553-559. doi:10.1002/hup.1152	Unfocused
7.	Mihaljevic Peles A, Bozina N, Sagud M, Rojnic Kuzman M, Lovric M. MDR1 gene polymorphism: therapeutic response to paroxetine among patients with major depression. Prog Neuropsychopharmacol Biol Psychiatry. 2008;32(6):1439-1444. doi:10.1016/j.pnpbp.2008.03.018	Unfocused
8.	Storch CH, Nikendei C, Schild S, Haefeli WE, Weiss J, Herzog W. Expression and activity of P-glycoprotein (MDR1/ABCB1) in peripheral blood mononuclear cells from patients with anorexia nervosa compared with healthy controls. Int J Eat Disord. 2008;41(5):432-438. doi:10.1002/eat.20519	Unfocused
9.	Kato M, Fukuda T, Serretti A, et al. ABCB1 (MDR1) gene polymorphisms are associated with the clinical response to paroxetine in patients with major depressive disorder. Prog Neuropsychopharmacol Biol Psychiatry. 2008;32(2):398-404. doi:10.1016/j.pnpbp.2007.09.003	Unfocused
10.	Mihaljević-Peles A, Sagud M, Bozina N, Zivković M. Pharamacogenetics and antidepressant treatment in integrative psychiatry perspective. Psychiatr Danub. 2008;20(3):399-401.	Review
11.	Altinoz MA, Gedikoglu G, Sav A, et al. Medroxyprogesterone acetate induces c6 glioma chemosensitization via antidepressant-like lysosomal phospholipidosis/myelinosis in vitro. Int J Neurosci. 2007;117(10):1465-1480. doi:10.1080/00207450701540062	Duplicated
12.	Ehret MJ, Levin GM, Narasimhan M, Rathinavelu A. Venlafaxine induces P-glycoprotein in human Caco-2 cells. Hum Psychopharmacol. 2007;22(1):49-53. doi:10.1002/hup.820	Unfocused
13.	Weber CC, Kressmann S, Ott M, Fricker G, Müller WE. Inhibition of P-glycoprotein function by several antidepressants may not contribute to clinical efficacy. Pharmacopsychiatry. 2005;38(6):293-300. doi:10.1055/s-2005-916184	Unfocused

Table S4. PRISMA 2020 checklist.

Section and Topic	Item #	Checklist item	Location where item is reported
**TITLE**			
Title	1	Identify the report as a systematic review.	Pag. 1
**ABSTRACT**			
Abstract	2	See the PRISMA 2020 for Abstracts checklist.	Pag. 1
**INTRODUCTION**			
Rationale	3	Describe the rationale for the review in the context of existing knowledge.	Pag. 2
Objectives	4	Provide an explicit statement of the objective(s) or question(s) the review addresses.	Pag. 2
**METHODS**			
Eligibility criteria	5	Specify the inclusion and exclusion criteria for the review and how studies were grouped for the syntheses.	Pag. 3
Information sources	6	Specify all databases, registers, websites, organisations, reference lists and other sources searched or consulted to identify studies. Specify the date when each source was last searched or consulted.	Pag. 3
Search strategy	7	Present the full search strategies for all databases, registers and websites, including any filters and limits used.	Pag. 3
Selection process	8	Specify the methods used to decide whether a study met the inclusion criteria of the review, including how many reviewers screened each record and each report retrieved, whether they worked independently, and if applicable, details of automation tools used in the process.	Pag. 3
Data collection process	9	Specify the methods used to collect data from reports, including how many reviewers collected data from each report, whether they worked independently, any processes for obtaining or confirming data from study investigators, and if applicable, details of automation tools used in the process.	Pag. 3
Data items	10a	List and define all outcomes for which data were sought. Specify whether all results that were compatible with each outcome domain in each study were sought (e.g. for all measures, time points, analyses), and if not, the methods used to decide which results to collect.	Pag. 3
	10b	List and define all other variables for which data were sought (e.g. participant and intervention characteristics, funding sources). Describe any assumptions made about any missing or unclear information.	Pag. 3
Study risk of bias assessment	11	Specify the methods used to assess risk of bias in the included studies, including details of the tool(s) used, how many reviewers assessed each study and whether they worked independently, and if applicable, details of automation tools used in the process.	Pag. 3-4 Suppl
Effect measures	12	Specify for each outcome the effect measure(s) (e.g. risk ratio, mean difference) used in the synthesis or presentation of results.	NA
Synthesis methods	13a	Describe the processes used to decide which studies were eligible for each synthesis (e.g. tabulating the study intervention characteristics and comparing against the planned groups for each synthesis (item #5)).	Pag. 3
	13b	Describe any methods required to prepare the data for presentation or synthesis, such as handling of missing summary statistics, or data conversions.	Pag. 3-4
	13c	Describe any methods used to tabulate or visually display results of individual studies and syntheses.	Pag. 3-4
	13d	Describe any methods used to synthesize results and provide a rationale for the choice(s). If meta-analysis was performed, describe the model(s), method(s) to identify the presence and extent of statistical heterogeneity, and software package(s) used.	Pag. 3-4
	13e	Describe any methods used to explore possible causes of heterogeneity among study results (e.g. subgroup analysis, meta-regression).	NA
	13f	Describe any sensitivity analyses conducted to assess robustness of the synthesized results.	NA
Reporting bias assessment	14	Describe any methods used to assess risk of bias due to missing results in a synthesis (arising from reporting biases).	Pag. 3,5
Certainty assessment	15	Describe any methods used to assess certainty (or confidence) in the body of evidence for an outcome.	NA
**RESULTS**			
Study selection	16a	Describe the results of the search and selection process, from the number of records identified in the search to the number of studies included in the review, ideally using a flow diagram.	Pag. 5
	16b	Cite studies that might appear to meet the inclusion criteria, but which were excluded, and explain why they were excluded.	Suppl.
Study characteristics	17	Cite each included study and present its characteristics.	Pag. 5-13 Suppl.
Risk of bias in studies	18	Present assessments of risk of bias for each included study.	Pag.5-6

Section and Topic	Item #	Checklist item	Location where item is reported
			Suppl.
Results of individual studies	19	For all outcomes, present, for each study: (a) summary statistics for each group (where appropriate) and (b) an effect estimate and its precision (e.g. confidence/credible interval), ideally using structured tables or plots.	Suppl.
Results of syntheses	20a	For each synthesis, briefly summarise the characteristics and risk of bias among contributing studies.	Pag. 5-13 Suppl.
	20b	Present results of all statistical syntheses conducted. If meta-analysis was done, present for each the summary estimate and its precision (e.g. confidence/credible interval) and measures of statistical heterogeneity. If comparing groups, describe the direction of the effect.	Pag. 5-13
	20c	Present results of all investigations of possible causes of heterogeneity among study results.	NA
	20d	Present results of all sensitivity analyses conducted to assess the robustness of the synthesized results.	NA
Reporting biases	21	Present assessments of risk of bias due to missing results (arising from reporting biases) for each synthesis assessed.	Pag.5-6 Suppl.
Certainty of evidence	22	Present assessments of certainty (or confidence) in the body of evidence for each outcome assessed.	Suppl.
**DISCUSSION**			
Discussion	23a	Provide a general interpretation of the results in the context of other evidence.	Pag. 13-16
	23b	Discuss any limitations of the evidence included in the review.	Pag. 15
	23c	Discuss any limitations of the review processes used.	Pag. 15
	23d	Discuss implications of the results for practice, policy, and future research.	Pag. 15
**OTHER INFORMATION**			
Registration and protocol	24a	Provide registration information for the review, including register name and registration number, or state that the review was not registered.	Pag. 4
	24b	Indicate where the review protocol can be accessed, or state that a protocol was not prepared.	Pag. 4
	24c	Describe and explain any amendments to information provided at registration or in the protocol.	NA
Support	25	Describe sources of financial or non-financial support for the review, and the role of the funders or sponsors in the review.	Pag. 17
Competing interests	26	Declare any competing interests of review authors.	Pag. 17
Availability of data, code and other materials	27	Report which of the following are publicly available and where they can be found: template data collection forms; data extracted from included studies; data used for all analyses; analytic code; any other materials used in the review.	NA

Table S5. Customization of the OHAT RoB tool to the research topic. Questions 3 and 4 of the OHAT RoB tool do not apply to *in vitro* studies and have not been considered. However, the original numbering of OHAT RoB has been kept.

**Bias domain**	**Questions**	**1**	**2**	**3**	**4**	**5**	**6**	**7**	**8**	**9**	**10**	**11**
Selection	1. Was administered dose or exposure level adequately randomized?	-	-	-	+	-	-	-	-	+	+	-
	2. Was allocation to study groups adequately concealed?	-	-	-	-	-	-	-	-	-	-	-
Performance	5. Were experimental conditions identical across study groups?	+	+	+	++	++	+	++	++	++	++	++
	6. Were the research personnel and human subjects blinded to the study group during the study?	-	-	-	-	-	-	-	-	-	-	-
Attrition	7. Were outcome data complete without attrition or exclusion from analysis?	+	+	++	++	+	++	++	++	++	++	++
Detection	8. Can we be confident in the exposure characterization?	+	+	+	+	++	+	+	+	++	++	++
	9. Can we be confident in the outcome assessment?	+	++	+	++	++	++	+	++	+	+	++
Selective Reporting	10. Were all measured outcomes reported?	+	+	++	++	+	++	++	++	+	+	++
Other Sources	11. Were there no other potential threats to internal validity (e.g., statistical methods were appropriate and researchers adhered to the study protocol)?	-	+	++	++	+	++	++	++	+	+	++

Key: ++ Definitely Low risk of bias; + Probably Low risk of bias; - Probably High risk of bias; -- Definitely High risk of bias

Figure S1. Customization of the OHAT RoB tool for the research topic (questions 3 and 4 not applicable to in vitro studies).

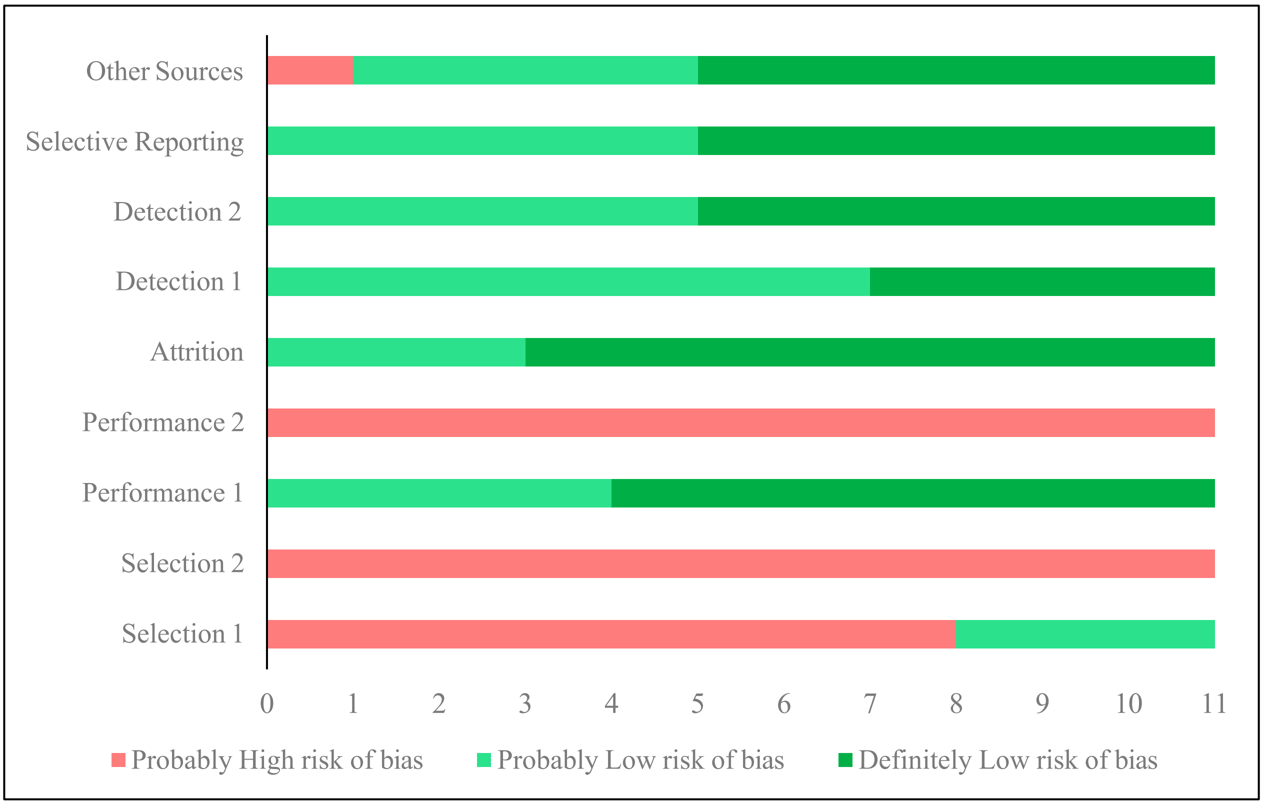

ID	Table S6. Full list of references of included studies in chronological order.

1	Fan D, Poste G, Obrian C, et al. Chemosensitization of murine fibrosarcoma cells to drugs affected by the multidrug resistance phenotype by the antidepressant trazodone - an experimental-model for the reversal of intrinsic drug-resistance. *Int J Oncol*. 1992;1(7):735-742.
2	Szabó D, Szabó G Jr, Ocsovszki I, Aszalos A, Molnár J. Anti-psychotic drugs reverse multidrug resistance of tumor cell lines and human AML cells ex-vivo. *Cancer Lett*. 1999;139(1):115-119. doi:10.1016/s0304-3835(99)00020-8
3	Peer D, Dekel Y, Melikhov D, Margalit R. Fluoxetine inhibits multidrug resistance extrusion pumps and enhances responses to chemotherapy in syngeneic and in human xenograft mouse tumor models. *Cancer Res*. 2004;64(20):7562-7569. doi:10.1158/0008-5472.CAN-03-4046
4	Argov M, Kashi R, Peer D, Margalit R. Treatment of resistant human colon cancer xenografts by a fluoxetine-doxorubicin combination enhances therapeutic responses comparable to an aggressive bevacizumab regimen. *Cancer Lett*. 2009;274(1):118-125. doi:10.1016/j.canlet.2008.09.005
5	Palmeira A, Rodrigues F, Sousa E, Pinto M, Vasconcelos MH, Fernandes MX. New uses for old drugs: pharmacophore-based screening for the discovery of P-glycoprotein inhibitors. *Chem Biol Drug Des*. 2011;78(1):57-72. doi:10.1111/j.1747-0285.2011.01089.x
6	Zhang Y, Zhou T, Duan J, Xiao Z, Li G, Xu F. Inhibition of P-glycoprotein and glutathione S-transferase-pi mediated resistance by fluoxetine in MCF-7/ADM cells. *Biomed Pharmacother*. 2013;67(8):757-762. doi:10.1016/j.biopha.2013.04.012
7	Drinberg V, Bitcover R, Rajchenbach W, Peer D. Modulating cancer multidrug resistance by sertraline in combination with a nanomedicine. *Cancer Lett*. 2014;354(2):290-298. doi:10.1016/j.canlet.2014.08.026
8	Liu Y, Li T, Xu M, Che X, Jiang X. Fluoxetine enhances cellular chemosensitivity to cisplatin in cervical cancer. *Int J Clin Exp Med*. 2017;10:10521-10527.
9	Duarte D, Nunes M, Ricardo S, Vale N. Combination of Antimalarial and CNS Drugs with Antineoplastic Agents in MCF-7 Breast and HT-29 Colon Cancer Cells: Biosafety Evaluation and Mechanism of Action. *Biomolecules*. 2022;12(10):1490. Published 2022 Oct 16. doi:10.3390/biom12101490
10	Wang JQ, Liu XM, Zhu ZS, et al. Fluoxetine-Conjugated Platinum(IV) Prodrugs Targeting eEF2K and Conquering Multidrug Resistance against Triple-Negative Breast Cancer. *J Med Chem*. 2025;68(9):9661-9680. doi:10.1021/acs.jmedchem.5c00352
11	Özkaya Gül S, Şimşek B, Yıldız F, Aydemir E. Cytotoxic Effect of Escitalopram/Etoposide Combination on Etoposide-Resistant Lung Cancer. *Pharmaceuticals (Basel)*. 2025;18(4):531. Published 2025 Apr 5. doi:10.3390/ph18040531

Figure S2. Risk of bias assessment of the included animal studies using SYRCLE's assessment tool.

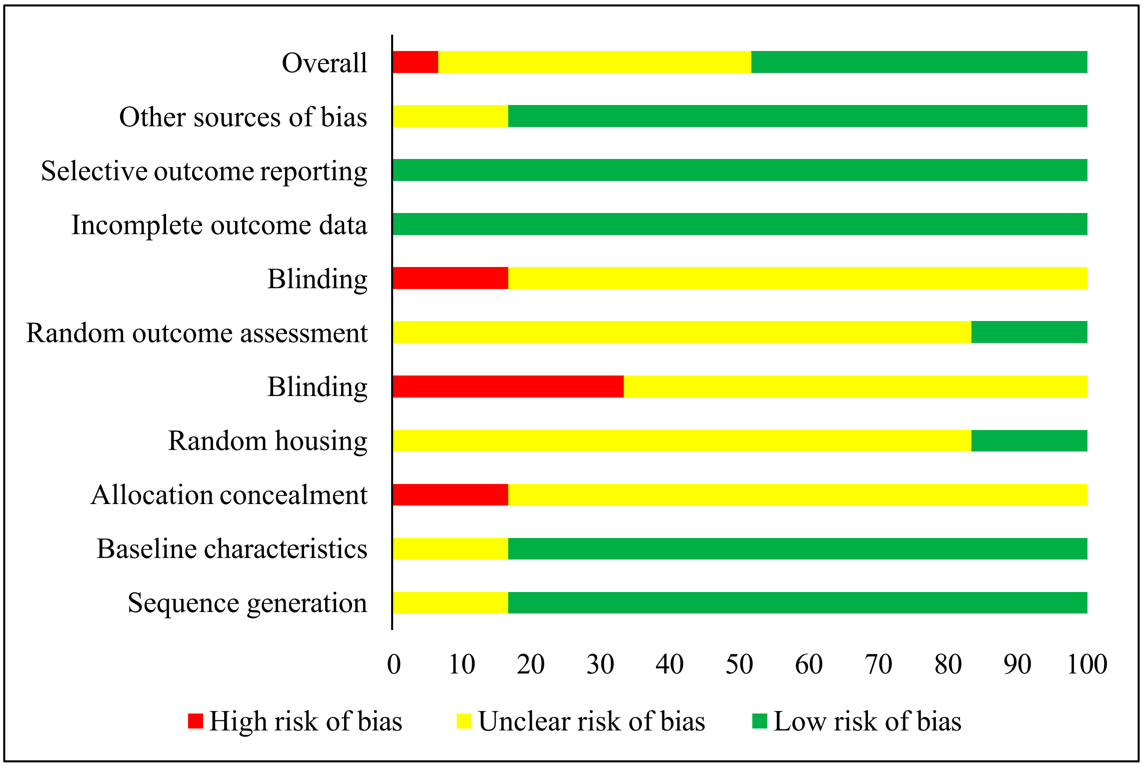

Table S7. Risk of bias assessment of the included animal studies using SYRCLE's assessment tool.

Type of bias	Domain	1	2	3	4	5	6	7	8	9	10	11
Selection	Sequence generation	NA	NA	?	+	NA	NA	+	+	+	+	NA
	Baseline characteristics	NA	NA	+	+	NA	NA	?	+	+	+	NA
	Allocation concealment	NA	NA	-	?	NA	NA	?	?	?	?	NA
Performance	Random housing	NA	NA	?	?	NA	NA	?	?	+	?	NA
	Blinding	NA	NA	-	?	NA	NA	?	?	-	?	NA
Detection	Random outcome assessment	NA	NA	?	?	NA	NA	?	+	?	?	NA
	Blinding	NA	NA	?	?	NA	NA	?	?	-	?	NA
Attrition	Incomplete outcome data	NA	NA	+	+	NA	NA	+	+	+	+	NA
Reporting bias	Selective outcome reporting	NA	NA	+	+	NA	NA	+	+	+	+	NA
Other	Other sources of bias	NA	NA	+	?	NA	NA	+	+	+	+	NA

Key: +, Low risk of bias; , Unclear risk of bias; , High risk of bias; NA Not Applicable (no in vivo study).

Section S2. Qualitative overview of the included studies

Design of the included studies

Five studies were exclusively in vitro, using cancer cell lines to examine the chemosensitizing effects of ADs on multidrug resistance (MDR) mechanisms[1–4]. Two investigations incorporated computational methodologies alongside laboratory validation. Palmeira et al. (2011)[5] performed in silico pharmacophore modeling and virtual screening to identify candidate ADs as modulators of P-glycoprotein, followed by functional ATPase and accumulation assays.

Five studies adopted a combined in vitro–in vivo design, integrating mechanistic cell-based experiments with animal tumor models to validate chemosensitization in living systems[6–10]. These studies used either xenograft or syngeneic mice to assess whether ADs enhanced drug uptake, delayed tumor growth, or improved survival following chemotherapy exposure.

Cellular models employed

The studies included in this review employed a diverse range of cellular systems designed to model different facets of multidrug resistance (MDR). Two major experimental frameworks were used: murine tumor cell lines and human cancer models, both incorporating drug-sensitive and chemoresistant counterparts to assess the ability of ADs to modulate cytotoxic responses.

Murine systems provided controlled platforms in which anthracycline resistance had been experimentally established, allowing a direct comparison between parental and MDR phenotypes. Examples include the UV-2237M fibrosarcoma and its adriamycin-resistant derivative[4], as well as the P388 leukemia wild-type and resistant lines23. These models demonstrated that exposure to selected ADs increased intracellular retention of chemotherapeutic agents, thereby reversing resistance mechanisms.

Human-derived systems broadened the biological relevance of these findings. These encompassed both immortalized cancer lines—such as breast (MCF-7/ADR)[1,3,6,7], colorectal (HT-29, HCT-116, HCT-15)[3,6–8], ovarian (OVCAR-8, NCI/ADR-RES, A2780/A2780cis)[9], cervical (HeLa)[6,10], lung (A549 and resistant derivatives)[2,6], and hepatocellular (HepG2)22—and hematologic malignancies , including leukemic models engineered or selected for transporter overexpression[5,11]. Within this group, Szabó et al. (1999)[11] uniquely analyzed primary leukemic blasts and peripheral blood lymphocytes from acute myeloid leukemia (AML) patients.

Across studies, MDR phenotypes were established through two mechanistically distinct strategies. In several models, resistance emerged following chronic exposure to cytotoxic agents such as adriamycin/doxorubicin, cisplatin or etoposide, mimicking clinically relevant drug-pressure–induced resistance[2,6,7]. In parallel, other systems[9,11] acquired MDR through genetic manipulation leading to overexpression of ATP-binding cassette (ABC) transporters, including P-gp, MRP1, MRP2, MRP7 and BCRP, thereby enabling precise interrogation of transporter-specific mechanisms of chemosensitization .

To evaluate the selectivity of AD–chemotherapy interactions and exclude nonspecific cytotoxicity, several investigations incorporated non-tumoral human cells as controls, including MRC-5 fibroblasts27, BEAS-2B bronchial epithelial cells21 and LO2 hepatocytes[2,3,6].

AD agents employed

The AD repertoire investigated across the included studies encompassed tricyclic ADs (TCAs), selective serotonin reuptake inhibitors (SSRIs), and a set of non-tricyclic psychoactive compounds with putative transporter-modulating activity.

Tricyclic ADs (TCAs) represented the earliest and most frequently tested class. Compounds such as imipramine, amitriptyline, desipramine, nortriptyline, trimipramine and doxepin, along with structurally related tetracyclic amoxapine and maprotiline, were assessed for their capacity to enhance intracellular accumulation of chemotherapeutics and inhibit efflux mediated by P-gp and related transporters[4,5,11]. These molecules were generally applied at low-to-moderate micromolar concentrations or clinically relevant plasma levels, enabling chemosensitization without direct cytotoxicity.

Subsequent research expanded the pharmacological scope to selective serotonin reuptake inhibitors (SSRIs), with most of the studies employing fluoxetine[1,3,6–8,10]. Sertraline[3,9] and escitalopram[2] were also employed as representative agents of this class. Wang et al. (2025)[6] designed a platinum(IV) prodrug in which fluoxetine is covalently attached to the platinum

scaffold, creating a single molecule that combines the structural features of an SSRI with a cytotoxic agent.

Beyond TCAs and SSRIs, Fan et al. (1992)[4] employed trazodone, a serotonin antagonist and reuptake inhibitor (SARI).

ADs were generally used at non-cytotoxic concentrations within the low-to-micromolar range. These concentration ranges were deliberately selected to remain below the IC_{50} thresholds, ensuring that any observed effects reflected modulation of MDR mechanisms rather than direct cytotoxicity.

Chemotherapeutic agents employed

Chemotherapeutic agents spanned multiple antineoplastic classes, predominantly anthracyclines, taxanes, platinum derivatives, vinca alkaloids and antimetabolites, all recognized substrates or modulators of ABC transporters. The most frequently used drugs were the anthracyclines doxorubicin[1,4,5,7–9,11] and daunorubicin[11]. Taxanes included paclitaxel[1,3,7], whereas vinca alkaloids comprised vincristine[4] and vinblastine[4,7]. Additional cytotoxic backbones included 5-fluorouracil and cytarabine[3,4,11], as well as actinomycin D[4], etoposide[2] and mitomycin C[4,7], used in early assays to test class-specificity of chemosensitization. Platinum-based agents included cisplatin, oxaliplatin and carboplatin[6,7,10], while pegylated liposomal doxorubicin (DOXIL) was used in xenograft models[9]. One study incorporated the TAD/COAP leukemia regimen[11] and bevacizumab (BEV)[8] as non-classical comparators. The most recent advancement in this research trajectory involved substituting conventional chemotherapeutic agents with a fluoxetine–Pt(IV) hybrid prodrug[6], thereby unifying anticancer activity and chemosensitization within a single molecular entity.

Dosing strategies across studies were consistently anchored to IC_{50} thresholds of the respective chemotherapeutic agents, with concentrations deliberately maintained below or around the half-maximal inhibitory range to prevent complete cytotoxicity. This approach ensured that experimental conditions preserved measurable cell viability, allowing investigators to attribute observed enhancements in anticancer activity to modulation of drug efflux and transporter function by ADs, rather than to nonspecific additive toxicity or supra-pharmacological exposure. By positioning both ADs and chemotherapeutics below their IC_{50} values, the included studies isolated chemosensitizing effects as mechanistically distinct from direct cell killing, thereby reinforcing the causal link between transporter inhibition and restoration of drug susceptibility.

Methods and techniques employed

Across the included investigations, cytotoxicity and viability were predominantly assessed in vitro using assays such as the 3-(4,5-dimethylthiazol-2-yl)-2,5-diphenyltetrazolium bromide (MTT) assay, the 2,3-bis-(2-methoxy-4-nitro-5-sulfophenyl)-2H-tetrazolium-5-carboxanilide (XTT) assay, the sulforhodamine B (SRB) assay, the Cell Counting Kit-8 (CCK-8) assay, trypan blue exclusion and neutral red uptake, which were used to quantify growth inhibition and shifts in half-maximal inhibitory concentrations (IC_{50}) under single-agent and combination conditions[1–6,8,9,11]. In primary leukemic blasts and peripheral blood lymphocytes, Szabó et al. (1999)[11] evaluated treatment effects by combining a [^{125}I]iododeoxyuridine ([^{125}I]IdUrd) DNA synthesis assay with trypan blue viability, thereby capturing both proliferation inhibition and cell survival in ex vivo human samples. Palmeira et al. (2011)[5] complemented in silico analyses with SRB-based growth inhibition assays in K562Dox cells to determine doxorubicin chemosensitization.

Similarly, in studies that incorporated animal models[4,6–9], cell-based viability assays were systematically paired with in vivo readouts such as tumor volume, survival and pharmacokinetic or biodistribution endpoints, ensuring that AD-mediated chemosensitization was captured at both cellular and whole-tumor levels.

Drug accumulation and efflux dynamics were examined using fluorescent or radiolabeled substrates such as rhodamine-123 (R-123), intrinsic DOX/ADR fluorescence, [^{3}H]-vinblastine and [^{3}H]-paclitaxel, quantified primarily through flow cytometry or fluorimetry[3,4,6–9,11].

Multiple investigations measured transporter biology by assessing the expression of P-gp, MRP1, MRP2, MRP7) and BCRP through Western blotting and flow cytometry, while RT-PCR/qRT-PCR quantified transcriptional levels of MDR1 and related genes[1,3,4,6,9]. P-gp ATPase assays, along with in silico pharmacophore modeling, molecular docking and MM-GBSA calculations, were used to characterize transporter–drug interactions19.

Several studies interrogated apoptotic and stress-related pathways using Annexin V–FITC/PI staining, JC-1 mitochondrial membrane potential assays ($\Delta\Psi m$), caspase-3 ELISA, and immunodetection of markers including cleaved PARP, Bcl-2, ROS, LC3, Beclin-1, p62, NF-κB p65, PTEN, ATM, RAD51 and PD-L1[2,3,6,9,10].

References

1. *Zhang Y, Zhou T, Duan J, Xiao Z, Li G, Xu F. Inhibition of P-glycoprotein and glutathione S-transferase-pi mediated resistance by fluoxetine in MCF-7/ADM cells. Biomed Pharmacother. 2013;67(8):757-762.*
2. *Özkaya Gül S, Şimşek B, Yıldız F, Aydemir E. Cytotoxic effect of escitalopram/etoposide combination on etoposide-resistant lung cancer. Pharmaceuticals (Basel). 2025;18(4):531.*
3. *Duarte D, Nunes M, Ricardo S, Vale N. Combination of antimalarial and CNS drugs with antineoplastic agents in MCF-7 breast and HT-29 colon cancer cells: biosafety evaluation and mechanism of action. Biomolecules. 2022;12(10):1490.*
4. *Fan D, Poste G, Obrian C, et al. Chemosensitization of murine fibrosarcoma cells to drugs affected by the multidrug resistance phenotype by the antidepressant trazodone: an experimental model for the reversal of intrinsic drug resistance. Int J Oncol. 1992;1(7):735-742.*

5. *Palmeira A, Rodrigues F, Sousa E, Pinto M, Vasconcelos MH, Fernandes MX. New uses for old drugs: pharmacophore-based screening for the discovery of P-glycoprotein inhibitors. Chem Biol Drug Des. 2011;78(1):57-72.*
6. *Wang JQ, Liu XM, Zhu ZS, et al. Fluoxetine-conjugated platinum(IV) prodrugs targeting eEF2K and conquering multidrug resistance against triple-negative breast cancer. J Med Chem. 2025;68(9):9661-9680.*
7. *Peer D, Dekel Y, Melikhov D, Margalit R. Fluoxetine inhibits multidrug resistance extrusion pumps and enhances responses to chemotherapy in syngeneic and in human xenograft mouse tumor models. Cancer Res. 2004;64(20):7562-7569.*
8. *Argov M, Kashi R, Peer D, Margalit R. Treatment of resistant human colon cancer xenografts by a fluoxetine-doxorubicin combination enhances therapeutic responses comparable to an aggressive bevacizumab regimen. Cancer Lett. 2009;274(1):118-125.*
9. *Drinberg V, Bitcover R, Rajchenbach W, Peer D. Modulating cancer multidrug resistance by sertraline in combination with a nanomedicine. Cancer Lett. 2014;354(2):290-298.*
10. *Liu Y, Li T, Xu M, Che X, Jiang X. Fluoxetine enhances cellular chemosensitivity to cisplatin in cervical cancer. Int J Clin Exp Med. 2017;10:10521-10527.*
11. *Szabó D, Szabó G Jr, Ocsovszki I, Aszalos A, Molnár J. Antipsychotic drugs reverse multidrug resistance of tumor cell lines and human AML cells ex vivo. Cancer Lett. 1999;139(1):115-119.*
